# Review Cerebral Ischemic Tolerance and Preconditioning: Methods, Mechanisms, Clinical Applications, and Challenges

**DOI:** 10.3389/fneur.2020.00812

**Published:** 2020-09-18

**Authors:** Yulei Hao, Meiying Xin, Liangshu Feng, Xinyu Wang, Xu Wang, Di Ma, Jiachun Feng

**Affiliations:** Department of Neurology and Neuroscience Center, The First Hospital of Jilin University, Changchun, China

**Keywords:** cerebral ischemia, ischemic tolerance, ischemic preconditioning, local ischemic preconditioning, remote ischemic preconditioning, cross preconditioning

## Abstract

Stroke is one of the leading causes of morbidity and mortality worldwide, and it is increasing in prevalence. The limited therapeutic window and potential severe side effects prevent the widespread clinical application of the venous injection of thrombolytic tissue plasminogen activator and thrombectomy, which are regarded as the only approved treatments for acute ischemic stroke. Triggered by various types of mild stressors or stimuli, ischemic preconditioning (IPreC) induces adaptive endogenous tolerance to ischemia/reperfusion (I/R) injury by activating a multitude cascade of biomolecules, for example, proteins, enzymes, receptors, transcription factors, and others, which eventually lead to transcriptional regulation and epigenetic and genomic reprogramming. During the past 30 years, IPreC has been widely studied to confirm its neuroprotection against subsequent I/R injury, mainly including local ischemic preconditioning (LIPreC), remote ischemic preconditioning (RIPreC), and cross preconditioning. Although LIPreC has a strong neuroprotective effect, the clinical application of IPreC for subsequent cerebral ischemia is difficult. There are two main reasons for the above result: Cerebral ischemia is unpredictable, and LIPreC is also capable of inducing unexpected injury with only minor differences to durations or intensity. RIPreC and pharmacological preconditioning, an easy-to-use and non-invasive therapy, can be performed in a variety of clinical settings and appear to be more suitable for the clinical management of ischemic stroke. Hoping to advance our understanding of IPreC, this review mainly focuses on recent advances in IPreC in stroke management, its challenges, and the potential study directions.

## Introduction

Cerebrovascular disease is one of the main diseases that lead to human death and disability worldwide, which endangers the health and life of middle-aged and elderly people (1, 2). About 795,000 new or recurrent cerebrovascular diseases occur every year, among which 87% are ischemic cerebrovascular diseases (1). Ischemic cerebrovascular diseases are mainly cerebral infarctions caused by the interruption of cerebral blood flow due to thrombus, embolism, or other reasons in the cerebral blood vessels, resulting in energy metabolism depletion and disorders of ion homeostasis; membrane depolarization; inhibition of high-energy phosphates; cellular potassium efflux; and water, sodium, and chloride influx, followed by a subsequent host cascade of mechanisms, including excitotoxicity, calcium overload, oxidative/nitrative stress, free radical generation, apoptosis, and inflammation, which trigger irreversible brain injury (3). Therein, neurons, glial cells, endothelial cells, and their interconnections are severely damaged and trigger each other in a positive feedback loop and eventually lead to damage and death of nerve cells.

The currently approved treatments for acute cerebral ischemia include the venous injection of thrombolytic tissue plasminogen activator (tPA) within 4.5 h and thrombectomy within 24 h after the appearance of neurological symptoms, which, however, could inevitably induce ischemia/reperfusion (I/R) injury (1, 4). However, the narrow therapeutic window and potential side effects limit their clinical application. In the past few decades, researchers have also carried out a large number of experimental studies on cerebral ischemic neuroprotective agents, but the results were not as expected when these agents were used clinically. Therefore, it is necessary to further fully understand the complex cascade mechanism of its pathological process and advance cost-effective and neuroprotective strategies for ischemic stroke treatment.

Being challenged by nutrient and oxygen deprivation, the brain starts potent endogenous defensive mechanisms against the complex deleterious cascade mechanism, which is also an underlying mechanism leading to irreversible lethal ischemic injury (5). Thus, the endogenous defensive mechanisms that protect the brain against ischemic stimuli and recovers from injury become an increasing hot spot. Ischemic preconditioning (IPreC), referring to a non-injurious and sublethal ischemic insult, can mediate complex endogenous protective mechanisms and provide ischemic tolerance and potent protection against a subsequent, otherwise lethal, ischemia (6, 7). IPreC is considered to be a potential and powerful neuroprotective mechanism that can cope with extreme metabolic stress, such as hypoxia or ischemia, which has aroused great interest in neurological experiments and clinical fields (8). In addition, studies have found a variety of physical and pharmacological stimuli can also induce ischemic tolerance (9–13). Over the past decades, researchers have made significant progress in signifying the endogenous mechanisms of IPreC and in applying the above mechanisms of action to routine clinical practice.

## Understanding Cerebral Ischemic Preconditioning

In the past 30 years, ischemic tolerance, as an effective protective strategy for ischemic diseases, has attracted wide attention and in-depth research. According to the time and process of sublethal ischemic injury and ischemic stroke, ischemic tolerance can be divided into the following three types: (1) ischemic preconditioning (IPreC), when sublethal ischemia insult is performed before ischemic disease; (2) ischemic perconditioning, when the ischemic stroke occurs and sublethal ischemia insults should be initiated at the same time; and (3) ischemic post-conditioning, when sublethal ischemia insult is implemented after the ischemic stroke. The above three methods may be involved in different endogenous protective mechanisms. In 1986, Murry et al. first described IPreC in myocardiac tissue, and most IPreC research focused on enhancing the resistance of the myocardium to subsequent fatal ischemic injury (13, 14). Studies have found that ischemic tolerance caused by IPreC is a common phenomenon and can be observed in various organs and tissues, such as heart, central nervous system (CNS), liver, retina, skeletal muscle, kidney, and intestine (15–17). Among them, tissues that are sensitive to hypoxia, such as myocardium, brain, and kidney, are the most promising targets for clinical application of IPreC (17–19).

Initially, an *in vitro* model of hippocampal slices was used to confirm the adaptability of rat brain tissue to anoxia, which caused wide concern in 1986 (20). In 1989, research showed that brief hypothermia could trigger neuroprotection (21). In 1990, Kitagawa et al. demonstrated that non-lethal ischemic insult could afford sufficient neuroprotection against neuronal death in the hippocampus CA1 region following subsequent lethal ischemic stress (22). In 1991, Kirino et al. also showed protective effect of non-lethal ischemic treatment in a global ischemia model of gerbils (23). During the late 1990s, a large number of research results consistently signified the IPreC-induced neuroprotection against lethal ischemic injury in focal and global cerebral ischemia of different animals (24, 25). One of the disadvantages of IPreC, which cannot be ignored, is that IPreC is capable of leading to serious damage with only small changes in the timing, durations, and location of sublethal ischemic insults (26). Therefore, researchers work tirelessly to find other safe and effective methods to safely induce ischemic tolerance. Remote ischemic preconditioning (RIPreC) is a method in which cerebral ischemic tolerance is induced after a brief short-term I/R duration in distant organs or tissue (27). RIPreC was reported in the myocardium in 1993 (15) and was also confirmed to be neuroprotective against ischemic stroke in 2011 (28).

By now, numerous studies have shown that different inducers/stressors can mediate cerebral ischemic tolerance. In addition to classic mechanical IPreC methods, including local ischemic preconditioning (LIPreC) and RIPreC, there are still many types of endogenous or exogenous stimuli that can induce experimental animals, brain tissue slice and cell cultures develop ischemic tolerance, named cross-preconditioning, mainly including chronic hypoxia (29, 30), hyperoxic or oxidative stress (31), hypothermia or hyperthermia (32), pharmacological treatment and chemical agent application (33–37), cortical spreading depression (38, 39), electroacupuncture (40), sports activity (41), and others [(9, 11, 12); Table 1].

**Table 1:** Representative reported methods of cerebral ischemic preconditioning.

Classic mechanical preconditioning	Local ischemic preconditioning	*In vivo*	Focal ischemic preconditioning	Global-Global (20, 42–44) Global-Focal (45)
			Global ischemic preconditioning	Focal-Focal (46) Focal-Global (47)
		*In vitro*	Oxygen–glucose deprivation (48, 49)	
	Remote ischemic preconditioning	Kidney (50, 51), mesenteric artery (52), liver (53), limbs (54–56), etc.		
Cross-preconditioning	Hyperoxic/Hypoxic preconditioning (57–82)			
	Hypothermia and Hyperthermia preconditioning (58–62)			
	Chemical/Pharmacological preconditioning (63–77, 83–87)			
	Other methods, including cortical spreading depression (36, 37), electroacupuncture (88), ketogenic diet (89), exercise (90), transcranial low-level light therapy (91), etc.			

### Local Ischemic Preconditioning

LIPreC, one of the earliest mechanical methods for mediating IPreC, can induce cerebral protective tolerance to the subsequent prolonged lethal I/R injury by short-term I/R of the brain tissue (92). Transient ischemic attacks (TIA) and clinical practice of surgical protection in organ transplantation provides convincing evidence for the effectiveness of LIPreC (92–95).

TIAs are caused by thrombosis, embolism, or vasospasm in the blood supply vessels of the brain tissue, which temporarily and non-lethally block the blood supply to the target brain, but do not cause cerebral tissue infarction. TIAs have the same clinical symptoms as ischemic stroke but do not leave permanent neurological impairment (96). Some previous clinical research concerning stroke patients has shown that TIAs can mediate the protective ischemic tolerance of brain tissue to subsequent lethal cerebral ischemia to a certain extent. The results of a case-control study of stroke patients in Germany suggest that the occurrence of previous TIAs can reduce the severity of subsequent lethal ischemic strokes (97). Another study compared the clinical data of stroke patients with or without prior TIAs, and the results show that ipsilateral TIA that lasted for 10–20 min before cerebral ischemia can produce significant ischemic protection (98). Wegener et al. demonstrated that patients with prior TIAs showed smaller initial cerebral diffuse injury and smaller final brain infarct volume within 12 h after the onset of stroke; however, there was no significant difference in the size and severity of hypoperfusion (99). To further explore the protective effects and possible neuroprotective mechanisms of IPreC on subsequent stroke, more clinical and experimental research needs to be conducted (95, 100, 101). Paradoxically, a cohort study involving more than 1,000 stroke patients showed that there was no correlation between prior TIA attacks and disability rates due to subsequent strokes, and the proportion of neurological impairment in stroke patients occurring 1–7 days after TIA onset was even higher (102). Individual heterogeneity among patients and diversity of the etiology of TIAs and subsequent stroke may be two important reasons for the discrepant results of the above studies.

As a means of surgical protection, LIPreC has been studied for many years and made great progress in organ transplantation, trauma, aneurysm surgery, and other fields. It was evaluated whether ischemic tolerance induced by occlusion at the proximal artery for 2 min could reduce the brain tissue damage caused by the subsequent clipping of cerebral aneurysm during the operation in 12 patients with aneurysmal subarachnoid hemorrhage (103). The results showed that the baseline gas pressure and pH value of the two groups were similar, but the decrease of oxygen pressure and pH value in the IPreC group was slower than that in the control group (103). These results suggest that short-term occlusion of the proximal artery to induce ischemic tolerance may be an effective protective measure in complex cerebrovascular surgery.

#### Protection of Local Ischemic Preconditioning

According to previous research results, LIPreC mainly has two temporal protective tolerance windows (13). The first is the short-term window, which is also called the quick window, which usually appears a few minutes after pretreatment. The mechanism is formed by the changes of post-translational modification, and the window of protection is very short, which usually disappears after a few hours. The second window is the long-term window, also known as the delayed window, which usually appears within 1 day of pretreatment and lasts for a maximum of a week after preconditioning (8, 26). The mechanism mainly involves genetic reprogramming and epigenetic modifications, which ultimately involve changes in protein synthesis, and therein, the delay window has more important protective significance for the ischemic brain (104, 105). However, current research perspectives regard that there is usually an unprotected window with little or no neuroprotection between the first window and the second window (104). Recent research evidence suggests that repeated hypoxic preconditioning can even mediate brain tissue to produce a third protective window that lasts up to 8 weeks of which the underlying mechanism may be caused by epigenetic regulation (106, 107).

#### Methods of Local Ischemic Preconditioning

There are *in vivo* and *in vitro* means to locally precondition the ischemic brain (42, 43, 108). The *in vivo* methods mainly include focal IPreC and global IPreC, whether it is rapid or delayed ischemic tolerance (26, 42). In focal ischemic preconditioning, ischemic tolerance is caused by a single or several brief interruptions of the middle cerebral artery blood flow for several minutes and subsequent reperfusion in between (8). Global ischemic preconditioning is caused by a single brief occlusion of the two common carotid arteries that supply the forebrain tissue or all the four cerebral vessels, usually <5 min (8, 42). Within the subsequent protective time window of sufficient ischemic tolerance, the lethal ischemic insults of a longer duration were established thereafter. It should be noted that focal and global ischemia can produce interactive ischemic tolerance, so different preconditioning modes by varying durations of focal and global ischemia insults were established (8). There are mainly four different experimental ischemic duration modes: (1) global ischemic preconditioning through two- or four-vessel occlusion before final global ischemia (22, 44–46), (2) global ischemic preconditioning by four-vessel occlusion before permanent focal ischemia (47), (3) focal ischemic preconditioning by transient middle cerebral artery occlusion (MCAO) followed by permanent MCAO in rats (109), (4) focal ischemic preconditioning induced by unilateral MCAO followed by global ischemia (25, 110). It should be noted that not all combinations of ischemic time and reperfusion time can trigger ischemic tolerance to play a protective role in brain tissue. Ischemic tolerance can be induced by 3–5 min of focal ischemia and at least 5 min of reperfusion but cannot be induced by 1–2 min of focal ischemia (111, 112). Single ischemia-reperfusion treatment can effectively induce ischemic tolerance, but repeated transient ischemia-reperfusion treatment also has an obvious protective effect (17, 48, 49, 113). The safe and effective time collocation of ischemia and reperfusion still needs a lot of follow-up experimental research.

There are currently a variety of *in vitro* IPreC methods, of which oxygen-glucose deprivation (OGD) is the most widely used model (114). The OGD model established by neural cell line culture and brain tissue is a particularly useful tool for studying its tolerance mechanism under *in vitro* conditions and is beneficial to exclude the influence of systemic factors after stroke (114, 115). Studies have shown that, after being exposed to OGD pretreatment for a short period of time, the cortical culture of murine brain tissue was subsequently exposed to OGD for a longer time, and the death of cortical neurons was reduced by 30–50%, but this protective effect only lasts between 7 and 72 h (115).

#### Mechanisms of Local Ischemic Preconditioning

In most cases, various forms of pretreatment stimulation can trigger endogenous protection or regeneration mechanisms through various signaling molecules and mechanism pathways, thereby generating protective ischemic tolerance from subsequent cerebral ischemic injury (26). IPreC involves a complex and interacting protective cascade mechanism, which can effectively reduce nerve cell damage after subsequent lethal stimuli. The induction of protective ischemic tolerance by IPreC is usually specific to the applied preconditioning stimuli and also related to the applied durations (5, 8, 26, 105). Existing research results support the view that energy depletion, ion disorders, excitatory amino acid toxicity, and lactic acid generation can occur immediately after an appropriate non-lethal ischemic attack, thereby activating the transcription factors and promoting protein synthesis that plays a protective role in neural cells (5, 116–121). Moreover, microarray analysis identified that gene expression is reprogrammed (122), and epigenetic modifications and post-transcription translation levels also changed significantly (121), mediating the protective phenotype so that neurons become responsive to the subsequent lethal ischemic attack.

##### Hypoxia inducible factor

Hypoxia conditions can trigger the generation of hypoxia-inducible factor-1α (HIF-1α), which is an oxygen-sensitive transcription factor. It can be significantly upregulated after cerebral ischemia and then participate in regulating the expression of various genes, thereby triggering various physiological responses (123–129). IPreC has different effects on the expression of HIF-1α in different cells, and it can increase the expression of HIF-1α in neurons quickly and transiently but slowly and continuously in astrocytes (130). It has been reported that the inhibition of prolylhydroxylase 2 (PHD2), which can promote the degradation of HIF-1α depending on the oxygen level, can promote the expression of neuronal HIF-1α, and is not involved in the induction of ischemic tolerance (131). However, astrocytic HIF-1α was independent of PHD2, which allows astrocytes to cause long-lasting HIF-1α expression and was rather essential for induction of ischemic tolerance efficiently (132). IPreC can also attenuate neuronal death induced by ischemic insults in the gerbil hippocampal CA1 region (CA1) throughout upregulation of HIF-1α, which enhances vascular endothelial growth factor (VEGF) expression and nuclear factor-kappa B (NF-κB) activation (125).

##### Glutamate pathway

Glutamate excitotoxicity is a chief mechanism of action in nerve cell injury following stroke (133, 134). After ischemia, lack of oxygen supply can decrease adenosine triphosphate (ATP) levels and increase glutamate levels significantly, which can overactivate the N-methyl-D-aspartate (NMDA) receptor and result in excessive calcium influx, impaired synaptic plasticity, and accumulation of glutamate (133, 135, 136). In neuronal cortical cultures, glutamate preconditioning can mediate ischemic tolerance while antagonists of NMDA and α-amino-3-hydroxy-5-methyl-4-isoxazole propionic acid (AMPA) receptor can eliminate the protective effect of glutamate pretreatment preconditioning (104, 106). Appropriate moderate activation of NMDA receptors is necessary to induce ischemic tolerance; studies have shown that this mechanism may involve NF-κB and tumor necrosis factor-α (TNF-α) pathways (137–139). It has been reported that activation of the NMDA receptor can inhibit the activation of stress-activated c-Jun N-terminal kinase (JNK) and protein kinase B (Akt), promote the activation of extracellular signal-regulated kinase (ERK1/2), and regulate the activity of normal cyclin adenosine monophosphate (cAMP) responsive element binding (CREB) activity, which may be key signaling molecules that mediate protective tolerance (140–142).

Other studies have also shown the specific overexpression of GLT-1 in astrocytes (35–38). Studies have shown that p38 mitogen-activated protein kinase (p38 MAPK) is involved in the regulation of GLT-1 upregulation during the induction of ischemic tolerance.

Upregulation of astrocyte glutamate transporter-1 (GLT-1) was found to assist in inducing cerebral ischemic tolerance (143). Research evidence indicates that IPreC can inhibit the increase in extracellular glutamate after OGD, promote the uptake of extracellular glutamate, and increase the GLT-1 expression in rat cortical cultures (144). Other research results showed that astrocytes GLT-1 overexpression had significant neuroprotective effects (35–38). Research has shown that p38 mitogen-activated protein kinases (p38 MAPK) played an important role in the induction of GLT-1 upregulation during ischemic tolerance mediation (145). Gap junctions (GJs), composed of connexin 43 (Cx43), are found at the corresponding position where adjacent astrocytes contact each other and form channels that allow the molecular exchange and information transfer between astrocytes. Recent studies have shown that astrocytes can release excessive glutamate through GJs after ischemia, which may promote the expansion of the infarct core and the surrounding penumbra area (146, 147). It has been demonstrated that IPreC can block GJs between astrocytes and decrease the extracellular glutamate content and reduce reactive oxygen species (ROS) injury in astrocytes, resulting in less neuronal damage (148).

##### Nitric oxide synthase

For both *in vivo* and *in vitro* models of IPreC, nitric oxide (NO) is a crucial member, but the exact mechanism of action remains unclear. In cortical cultures, activation of neuronal nitric oxide synthase (NOS) and subsequent other neuroprotective mechanisms may be an important part of a series of signaling cascades during OGD preconditioning (149). Anoxia preconditioning can protect rat hippocampal slices against the subsequent fatal anoxic injury, and interestingly, the NOS inhibitor (7-nitroindazole) can abolish the above protective effects (150). It was found that inducible NOS was involved in the neuroprotective tolerance induced by IPreC in both *in vivo* and *in vitro* conditions (141, 151–153). In addition, in endothelial NOS (eNOS) and neuronal NOS knockout mice, IPreC did not reduce the infarct volume of brain tissue in the focal ischemia model compared with the preconditioned wild-type counterpart (151). Recently, eNOS is considered to be a neurovascular protection mediator against vasospasm caused by subarachnoid hemorrhage, indicating that IPreC may also have a protective effect on other forms of stroke (154).

##### Immune system

Under an ischemia condition, a large number of inflammatory cells (such as microglia, lymphocytes, neutrophils, etc.) are recruited to the infarct area, mediating inflammatory damage to the brain tissue (155–158). The non-catalytic Toll-like receptor (TLR) signaling pathway can induce the transcription factor NF-κB to mediate the transcription of cytokines and chemokines by recognizing foreign signaling molecules, thereby initiating immune responses and establishing an inflammatory cascade, thereby causing secondary inflammation damage (158–161). The mechanism by which IPreC exerts neuroprotection is mainly through the promotion of anti-inflammatory molecules or inhibiting the expression of pro-inflammatory molecules. IPreC can reduce cerebral ischemic injury mainly through inhibiting TLR4/NF-κB signaling, enhancing interferon regulatory factor-dependent signaling, and inhibiting TLR4/myeloid differentiation factor 88 (MyD88) signaling, which resulted in an anti-inflammatory phenotype (162–164). It is reported that astrocytic TLR3 signaling plays an important role in IPreC-induced ischemic tolerance, which increases interferon secretion but decreased IL-6 secretion, resulting in suppression of the post-ischemic inflammatory response (160). Pérez-Pinzón et al. (165) reported that rapid IPreC can inhibit the activation of microglia after cerebral ischemia, mediating ischemic tolerance by exerting an anti-inflammatory effect. Chemokines promoting the migration of inflammatory cells play a vital role in the recruitment of inflammatory cells to ischemic brain tissue after ischemia, which direct the progression of inflammatory processes in stroke (165–167). The enhanced ability of IPreC-induced microglia to release chemokines can mediate the migration of leukocyte to protect the ischemic cells (168). In addition, IPreC can also mediate through the activation of cell surface chemokine receptor 2 to exert a neuroprotective effect (169).

##### Enzymes and receptors

sublethal cerebral ischemic insult can activate enzymes, which are the other group of proteins activated in IPreC. Studies have shown that the activation of Akt mediated by IPreC can negatively regulate the JNK signaling pathway (170). The physiological function of cyclooxygenase-2 (COX-2) is to promote the oxidation of arachidonic acid to prostaglandin, which plays an important role in the inflammatory damage after ischemia (171). Downregulation of COX-2 in IPreC was previously reported in gerbils, which means obstruction of the COX-2 pathway might be a therapeutic strategy in cerebral ischemia (172). IPreC can maintain or even increase the content of kynureic acid (KYNA) in pyramidal neurons of the hippocampal CA1 area, which indicates that IPreC-induced increase in KYNA expression is related to endogenous cerebral ischemic tolerance (173). The increase of adenosine after stroke is regarded to be neuroprotective, and the expression of adenosine receptors was increased after IPreC (174). Nakamura et al. reported that the adenosine A1 receptor may be related to rapid ischemic tolerance in rat focal ischemia (167). Adenosine kinase (ADK) can inhibit the expression of adenosine, and under-expression of cerebral ADK in transgenic mice can induce cortical protection, suggesting a promising target for developing a stroke therapy (174).

The physiological function of monocarboxylate transporter 4 (MCT4) is mainly involved in transferring monocarboxylates across phospholipid membranes, and recent studies have shown that it is related to cerebral ischemic injury. It was reported that IPreC elevated or maintained the expression of MCT4 in astrocytes and protected pyramidal neurons from ischemic damage in the ischemic CA1 region (175). It has been discovered that the accumulation of unfolded proteins in the endoplasmic reticulum (ER) lumen occurs in cerebral nerve cells during reperfusion following global or focal cerebral ischemia (176, 177). IPreC can inhibit ER stress-induced apoptosis and play a positive role in the protection of following focal cerebral I/R injury (178). Downregulation of adenosine monophosphate-activated protein kinase (AMPK) contributes toward delayed ischemic tolerance in a MACO model in male mice (179). The endogenous protection mechanisms of IPreC may also involve Na^+^/Ca^2+^ exchangers (NCXs). HIF-1 can increase the expression of NCX-1 and Akt signaling participating in mediating the expression of NCX-3 (180). Finally, many key players such as HIF-2α, silent information regulator protein 1, and CREB are also important parts of the preconditioning cascade (142, 181).

##### Autophagy and apoptosis

Autophagy, a process of internalizing and digesting damaged organelles or misfolded proteins to produce metabolic substrates for cell recycling, is believed to be one of the most important mechanisms in IPreC-induced tolerance (182–184). Studies have shown that ischemic injury can activate autophagy, and the activation of autophagy was related to neuroprotection (184). IPreC can activate autophagy, and 3-MA can abolish the IPreC-induced ischemic tolerance while rapamycin potentiates the IPreC-induced ischemic tolerance (185). In contrast to the neuroprotective function of autophagy, apoptosis mediated cell death in I/R injury, mainly through the caspase-3-dependent apoptosis pathway (186). Cerebral ischemia can mediate the overexpression of caspase-3, and IPreC can attenuate this overexpression during ischemia (187). Interestingly, three episodes of IPreC can activate autophagy and exert neuroprotection against apoptosis in the following ischemia (187). Therefore, apoptosis may be the therapeutic target, and inducing autophagy could be expected to become an important protective strategy. Recent studies have shown that bone morphogenetic protein 7 (BMP-7), an important regulator of cartilage and bone formation, is also involved in DNA synthesis and astrocyte differentiation in the rat midbrain (188). Studies have found that BMP-7 participates in IPreC-mediated endogenous protective mechanisms to induce ischemic tolerance (189). Inhibition of BMP-7 can attenuate apoptosis via inhibiting Bcl-2 and promoting cleaved caspase-3 (189). Moreover, BMP-7-mediated IPreC-induced neuroprotection may be through activation of p38 MAPK signaling pathway (190).

##### Energy metabolism

With the impaired delivery of glucose and oxygen in ischemia, the energy metabolism pattern of the brain changed from oxidative phosphorylation to excessive glycolysis, thereby promoting the rapid generation of ATP to meet energy expenditure (191). However, excessive glycolysis increased the production of lactic acid and ROS, which promoted the expansion of the ischemic penumbra (192, 193). IPreC can subdue post-ischemic hyperglycolysis and promote the utilization of β-hydroxybutyrate and provide a well-adapted metabolic background (192). A number of studies revealed that IPreC-related metabolic flexibility was associated with the downregulation of AMPK-mediated glucose transporter-1 and decreased the mRNA levels of nicotinamide adenine dinucleotide phosphate (NADPH) oxidase subunits (194).

Nuclear erythroid 2-related factor 2 (Nrf2) protein can reduce oxidative stress damage by upregulating the transcription of antioxidant-related protein under pathological conditions in astrocytes and may be a novel key player in nuclear-mitochondrial interaction and IPreC-mediated neuroprotection (195). Novel evidence indicates that Nrf2 plays an important role in oxidative phosphorylation supercomplex association, and the absence of Nrf2 can reduce IPreC-induced protection in astrocyte cultures (196, 197). Glucokinase (GK) and glucokinase regulatory protein (GKRP) may be involved in protecting neurons in the ischemic CA1 region (198). Certain studies have suggested that IPreC can significantly enhance the immunoreactivity of GK and GKRP in neurons of the CA1 region after 5 min of I/R, suggesting that an important mechanism for IPreC to promote the survival of neurons in CA1 region under ischemic conditions may be to maintain the expression of GK and GKRP (199).

##### Blood-brain barrier permeability

Blood-brain barrier (BBB) injury occurs soon after cerebral ischemia (200, 201), which allows infiltration of immune cells and inflammatory factors and leads to brain edema and hemorrhagic transformation (202). Tight junctions (TJs) and adherens junctions (AJs), located between adjacent endothelial cells of the BBB, is critical for BBB integrity (201). The transmembrane TJs and AJs are mainly composed of claudin-5 and cadherin 5, which are involved in regulating paracellular permeability (201). IPreC can maintain the BBB permeability by directly upregulating the TJs protein claudin 5 and the AJ protein cadherin 5 (109, 203) IPreC-induced cytokines, such as TNF-α and interleukin 1β (IL-1β), may affect BBB permeability (106). IPreC can mediate TJs and angiogenic factors levels via activating ERK1/2, implying an essential role of ERK1/2 in paracellular permeability in IPreC (106). Other studies showed that the underlying mechanisms of IPreC-mediated BBB protection involve VEGF, Nrf2, or inflammatory pathways (109, 201).

##### Electrophysiology

Research indicated that MCAO impaired the expression of long-term potentiation (LTP) in the hippocampal CA1 region (204). Moreover, the LTP magnitude remained at a relatively low level 4 months after permanent MCAO (57). IPreC can dramatically increase the neurotransmitter content in presynaptic neurons to promote basal synaptic transmission without obvious adverse effects on the LTP induction (205).

##### Transcriptional regulation

Recent research on IPreC-related ischemic tolerance has shed light on the modifications at gene level. HIF-1 can promote the transcription of survival genes after ischemic injury (123, 124). After hypoxic stress occurs, HIF-1α and HIF-1β combine to form a heterodimer HIF-1, and then the heterodimer binds to the hypoxic response element on the target genes to form a transcription complex that promotes the transcription of VEGF, erythropoietin (EPO), and glucose transporters (123, 170, 206). Studies have found that the HIF-2α subunit in astrocytes can promote the transcription of EPO mRNA (207); however, the HIF-2α-mediated transcriptional regulation signaling cascade in IPreC has not been deeply explored (207, 208). Transcription factor activator protein 1 (AP-1), composed of c-Jun and c-Fos, is a dimeric protein that functions in both neuroprotection and cell death (209). *In vivo* experiments indicate that the early activation of AP-1 and its enhanced binding affinity to DNA are involved in the neuroprotection induced by IPreC (210). In addition, JNK and Akt signaling pathways are also involved in IPreC-mediated transcriptional regulation (211, 212).

##### Genomic reprogramming

Clarifying the changes in gene expression profiles in neuroprotective phenotypes after ischemia can help to understand IPreC-induced tolerance (122). It is worth mentioning that IPreC-induced changes in gene expression profiles include not only upregulation of genes related to neuronal protection and regeneration and also inhibition of genes related to degenerative pathways during ischemic injury (122). Various regulatory molecules and processes, for example, transcription factors and a large number of post-translational modifications, jointly participate in the change of gene expression profiles induced by IPreC (213). GeneChip technology was used to identify genomic reprogramming induced by IPreC, and molecules involved in inducing ischemic tolerance, such as heat shock protein 70 (HSP-70) and transforming growth factor (TGF)-α were confirmed (213). DNA microarray technology, an efficient method for studying differential gene expression patterns of ischemic tolerance, were used to imply the genetic profile in IPreC-stimulated rat hippocampal slices and mouse cortex (118, 214), by which the genes related to cell survival and regeneration (such as HIF, insulin-like growth factor, etc.) are upregulated, and region-specific expression patterns can be observed apparently (215).

##### Epigenetic reprogramming

Existing evidence indicates that IPreC-mediated ischemic tolerance involves epigenetic reprogramming in neuronal cells, and microRNA (miRNA) plays an important role in this process. The results of Dharap and Centeno et al. showed that IPreC can mediate downregulation of 25 miRNAs and upregulation of 26 miRNAs in rat cortex, and 20 of these miRNAs maintained expression changes within 3 days (150). Atochin et al. (151) analyzed miRNAs transcription in mouse cortex and found that IPreC could upregulate miRNAs expession while lethal ischemia downregulated miRNA expression. The results of bioinformatic analysis of IPreC-mediated miRNAs expression profiles indicated that IPreC preferentially regulates miRNAs targeting transcriptional regulators (216). miR-132, which regulates methyl CpG binding protein 2 (MeCP2) expression, is one of the most notable downregulated miRNAs in the IPreC brain, and MeCP2 protein expression was increased while knocking out MeCP2 in transgenic mice abolished the IPreC-induced tolerance (151).

Although LIPreC does reduce reperfusion injury and related systemic consequences, its main disadvantages are direct injury by invasive operation on the target organ and mechanical damage to the main vascular structure. Moreover, it is difficult to predict when an ischemic event will occur, and therefore, it is not clinically possible to perform ischemic pretreatment within the effective protection time window. The disadvantages limit its clinical application.

### Remote Ischemic Preconditioning

RIPreC is a systemic protective strategy in which one or more cycles of ischemia and reperfusion durations in the target organ or tissues could confer protection against subsequent more severe ischemia insults in distant organs or tissues (217). RIPreC was first introduced in an animal cardiac study by Przyklenk et al. and first demonstrated in global cerebral ischemia model in rats (15). Experimental and clinical studies have demonstrated that RIPreC can induce cerebral tolerance to ischemic injury, augment cerebral perfusion status, reduce the risk of cerebral infarction, reduce TIA recurrence, promote the formation of cerebral collaterals, and increase the recovery rate (218–220). Subsequent studies confirmed that RIPreC decreases the infiltration of circulating leukocytes and provided a degree of cerebral protection (221). A large experimental animal model, has shown significantly better electroencephalogram results, better behavioral scores and histopathological scores in the RIPreC group 7 days post-operation (222). Additionally, patients undergoing carotid endarterectomy or patients undergoing elective cervical decompression surgery also benefitted from RIPreC (50, 219).

#### The Organs or Tissues for Performing Remote Ischemic Preconditioning

It has been evaluated that many organs or tissues, such as kidney (51, 52), mesenteric artery (53, 54), liver (55, 56), and limbs (223–225), can be considered as remote conditioned sites. Obviously, the most convenient and safest strategy of performing RIPreC should be on limbs when this preconditioning strategy is considered to be clinically used (28). Remote ischemic limb(s) preconditioning (limb-RIPreC), usually via binding the cuff to the distal limb(s) and inflating the cuff to a pressure that blocks the blood perfusion of the limb(s), has been used to effectively induce ischemic tolerance against subsequent I/R damage in animal experiments (223) and clinical trials over decades (28, 226). It has been found that acute ischemic stroke patients with a history of peripheral vascular disease (PVD) have significantly smaller infarct volumes, better clinical outcomes, and a lower mortality rate than those without PVD (227), also confirming the efficiency of limb-RIPreC.

#### Protection of Remote Ischemic Preconditioning

Similar to LIPreC, the results from clinical trials concerning the protection of RIPreC considered that there might be a first window (also called early, short window) and second window (also called later, prolonged window) of RIPreC (228). The neuroprotection of the first window occurs soon after pretreatment and is maintained for about 4 h (228). Ren et al. reported that limb-RIPreC via three cycles of 15 min femoral occlusion and reperfusion durations significantly reduced cerebral ischemic injury after 30 min of bilateral common carotid artery occlusion in rats (225), the same as the results of Wei et al. (229). Hu et al. (223) showed that performing limb-RIPreC 1 h before MCAO can obviously protect the subsequent cerebral ischemic injury in rats. Once believed to start 24 h after preconditioning (228), the later phase was corrected to start 12 h after preconditioning (225) and last for at least 48 h (228). Animal studies showed that RIPreC started 24 (230) and 48 h (225) prior to brain ischemia were both protective. Malhotra et al. also showed that RIPreC can't mediate protection when performed 48 h before brain ischemia onset (230).

#### Mechanisms of Remote Ischemic Preconditioning

**Figure 1:**
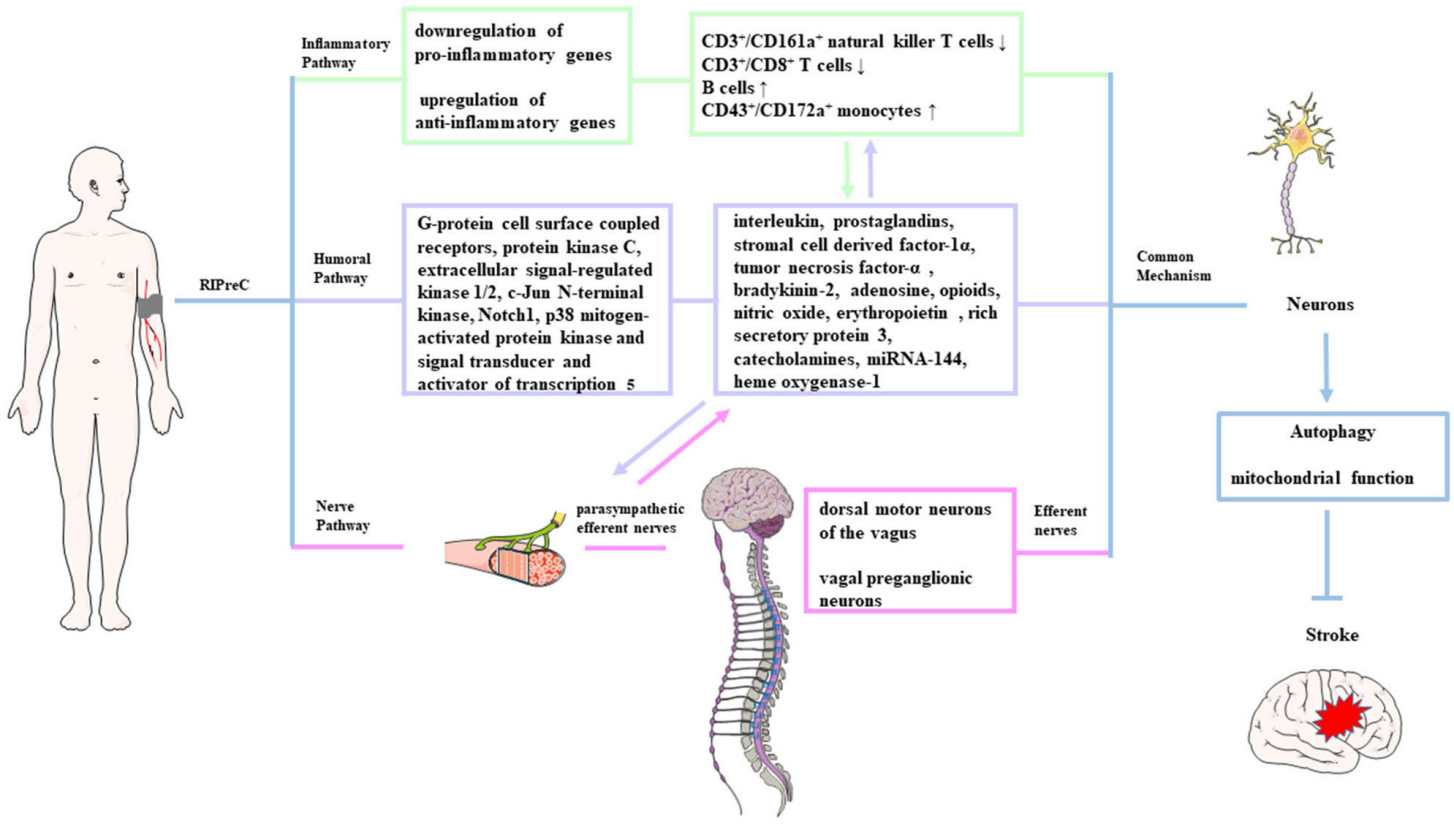
Possible mechanisms of remote ischemic preconditioning. The protective signals generated from the remote organ(s) are transmitted to the brain possibly through three pathways: neuronal (pink shading), humoral (purple shading), and immunological pathway (green shading). After being transmitted to the brain through the above three pathways, these signals induce autophagy activation and functional changes in the mitochondria, which is called a common signaling pathway.

At present, there is no unified view on the underlying mechanism of the protective effect transport to the target organ and the identity of the tolerance-inducing signal in the brain. There are three theories about the mechanism by which the protective effect mediated by ischemic preconditioning of distant organs or tissues is transferred to the brain tissue to protect the brain tissue against subsequent ischemic injury. It should be pointed out that these three theories are not mutually exclusive. The first theory holds that the transmission of neuroprotection is mainly mediated by various humoral factors in the blood circulation system. The second theory is that the autonomic nervous system plays an important role in the transmission of neuroprotection, and the last one is the immune pathway involving circulating cytokines, chemokines, and immune cells (231). After the protective effect is transmitted to the brain through the above three pathways, the ischemic tolerance is initiated through a common signaling pathway (Figure 1).

##### Humoral pathway

The humoral pathway is considered to exist because of two reasons: (1) a period of reperfusion is needed after ischemia in the preconditioned site during the RIPreC durations, suggesting that the protective factors need to be transported via circulation to the target organs from the conditioned site, and (2) the blood cross-circulation model identified the existence of protective humoral factors (232, 233). Similarly, Shimizu et al. (232) reported that treating the naive hearts with the blood from rabbits or humans after receiving RIPreC treatment can protect the naive recipient hearts against subsequent I/R injury, and the protective effect could be mediated by molecules <15 kDa. Research had identified that interleukin, prostaglandins, stromal cell-derived factor-1α, TNF-α, bradykinin-2, adenosine, opioids, NO, EPO, rich secretory protein 3, HSP, catecholamines, heme oxygenase-1, miRNA-144, etc. as possible candidate transfer factors (234, 235). The related signaling pathways or molecular targets of those humoral factors include G-protein cell surface coupled receptors, protein kinase C, Notch1, ERK1/2, JNK1/2, p38 MAPK, and signal transducer and activator of transcription 5 (STAT5) (16, 19, 236).

##### Nerve pathway

Increasing evidence from experiments about ischemic stroke confirms that a neuronal pathway is involved in the RIPreC protection transmission from the remote conditioned organ to the brain. Research inducing limb-RIPreC in MCAO rats by different methods demonstrated that a ganglion blocker (hexamethonium) could abrogate the neuroprotection of RIPreC (229, 237). Research by Mastitskaya et al. (237) suggests that the dorsal motor neurons of the vagus in the brain stem participated in RIPreC-mediated cardioprotection, and stimulation of these neurons can produce the same protective effect as RIPreC, indicating that the vagal preganglionic neurons are involved in the neural transmission mechanism. Activation of parasympathetic efferent nerves was neuroprotective, and vagal stimulation reduced cerebral infarct size (238). Animal research has reported that transection of the femoral nerve or spinal cord can abolish the protective effect of RIPreC in rabbits (239). Interestingly, the protective effects of RIPreC seem to be attenuated in patients with neuropathy, implicating the dependence on intact neural pathways (237).

However, the neuroprotective effect of RIPreC in mice was partially abolished when the femoral nerve or sciatic nerve was scathed, indicating the interaction of neural and humoral pathways (239). The neuronal mechanism may work in combination with the humoral pathway: The release of endogenous humoral factors from the conditioned organ first activated the afferent nerves in the conditioned organ and then activated the efferent nerves terminating at the target organ to induce neuroprotection. For example, limb-RIPreC allowed the preconditioned limb(s) to release autacoids that can activate neural pathways (240, 241). Animal studies showed that the release of humoral factors caused by limb-RIPreC would be abolished by femoral nerve transaction and the release of protective humoral factors can be induced by femoral nerve stimulation, indicating that the integrity of neural pathway is an important prerequisite for the production of humoral factors (240, 242, 243).

##### Inflammatory pathway

It has been reported that RIPreC has a systemic anti-inflammatory influence via suppression of proinflammatory genes in immune cells. Microarray analysis of blood samples showed downregulation of proinflammatory genes and upregulation of anti-inflammatory genes within 15 min of RIPreC and at 24 h after preconditioning, confirming that limb-RIPreC inhibited systemic inflammation (244). RIPreC can also reduce circulating neutrophil activation, inhibit the release of proinflammatory cytokines, and downregulate the expression of adhesion molecules in healthy adults (245). Recently, it was reported that RIPreC resulted in the reduction of CD3^+^/CD161a^+^ natural killer T cells and CD3^+^/CD8^+^ T cells and elevation in the percentage of B cells and CD43^+^/CD172a^+^ monocytes in circulation (246). Interestingly, the inflammation changes are associated with improved neurological functions, implying the direct evidence that the immune pathway participates in RIPreC-induced neuroprotection (246). It was suggested that the activation of immune cells and regulation of inflammatory genes were related to the release of endogenous opioids; therefore, the immunological pathway is also linked to the humoral pathway (247).

##### Final common mechanism

Based on the above evidence, we can conclude that the three neuroprotection transmission pathways via humoral, neural, and inflammatory mechanisms may be a relationship of mutual influence and promotion in RIPreC. After being transmitted to the brain, these protective signals had a final common pathway to induce ischemic tolerance. Autophagy can promote the degradation of damaged organelles and accumulated misfolded proteins to produce metabolic substrates to meet the energy consumption of the brain tissue after ischemic injury (248). There is a view that the autophagy regulation may be an important process in mediating the protective phenotype after neuroprotection was transmitted to the brain tissue. It was reported that limb-RIPreC activated autophagy and promoted the survival of neurons in rat spinal within 24 h after I/R injury (248). IPreC-mediated autophagy can promote the removal of harmful substances and inhibit the neurotoxic cascade to play a neuroprotective role, such as upregulation of B-cell lymphoma 2 (Bcl-2) and HSP-70, reduction in cytochrome c release, and inhibition of caspase-3 activity (249). Park et al. found that the activity of autophagy in the penumbra was enhanced, which was obviously related to the neuroprotective effect of RIPreC in cerebral ischemia (249). RIPreC can also increase the resistance of cells to ischemic insults by maintaining mitochondrial structural and functional integrity, decreasing mitochondrial degradation and consequently reducing apoptosis to achieve neuroprotection (250). The possible mechanism of autophagy in IPreC-mediated neuroprotection still needs further research.

Despite the efficiency of RIPreC, there are still issues that cannot be ignored. First, animal experiments tend to perform RIPreC operations on the hind limb(s) of experimental animals (78, 223, 225) while clinical trials would choose the upper limb(s) of the subjects (28, 218); it is still unknown whether the neuroprotective effects mediated by the precondition of the upper limbs and lower limbs (hind limbs) are different. Second, we still cannot confirm how many limbs and cycles of RIPreC should be involved to acquire the optimal efficiency because the number of restrained limbs, duration of ischemia in each cycle, and number of cycles of RIPreC were different among published articles. An animal study by Ren et al. (225) indicated that three cycles of limb preconditioning could induce stronger neuroprotection than two cycles. Therefore, increasing the number of preconditioned limbs and the cycles of preconditioning ischemia/reperfusion durations may enhance the protective effect of RIPreC.

### Cross-Preconditioning

For ethical reasons, it is not allowed to repeatedly block the cerebral arteries to mediate neuroprotection through ischemic preconditioning in humans; cross-preconditioning, induced by stressors or stimuli other than ischemia, such as hyperoxia/hypoxia, hypothermia or hyperthermia, chemical/pharmacological pretreatment, cortical spreading depression, electroacupuncture, excise, et al. (29, 38, 58, 105, 205), has gradually become a new research hot spot hoping to develop a new IPreC strategy in humans. Interestingly, research suggests that many stresses or stimuli seem to mediate protective tolerance through a common signaling pathway (105).

#### Hyperoxic and Hypoxic Preconditioning

In MCAO models of rats and mice, hyperoxia was shown to induce ischemic tolerance via genetic reprogramming (59). Regular normobaric hyperoxia (95% O_2_) treatment in rodents can reduce the infarct volume of brain tissue and exert obvious neuroprotective effects after cerebral ischemic injury (60). Hyperbaric oxygenation (HBO) was found to induce ischemic tolerance by reducing cellular apoptosis (61) and downregulating the expression of COX-2 in global ischemia model of rats and murine (60). Studies have shown that the NF-κB signaling pathway and its regulated target gene transcription could be activated by exposure to intermittent and long-term normobaric hyperoxia preconditioning conditions in rats (206). Repetitive hypoxic preconditioning induced sustained endogenous tolerance to stroke in a mouse model of stroke, characterized by upregulation of chemokine (C-X-C motif) ligand 12 at the BBB and inhibition of leukocyte–endothelial adherence, contributing to the endogenous, anti-inflammatory phenotype (62).

#### Hypothermia and Hyperthermia Preconditioning

Hypothermia treatment, widely used in surgical procedures and other clinical practice, is safe and practical based on evidence from randomized clinical trials (63, 64). In focal ischemic models, hypothermia preconditioning can confer rapid tolerance; however, merely increasing the duration of the preconditioning stimulation did not significantly enhance the neuroprotective effect (64). The underlying mechanism of the prolonged ischemic tolerance induced by hypothermia supposedly depends on protein synthesis (64, 65). Hyperthermia can also bring about protection against cerebral ischemia in rodent. Hyperthermia preconditioning conducted through a hot water bath in newborn rats with brain temperatures increased to 41.5–42°C, reduced neuronal damage after 2 h hypoxic ischemic insult (66). Additionally, exposure to hyperthermia (38–40°C) for 6 h protected astrocytes against cerebral I/R injury in mice (67).

#### Chemical/Pharmacological Preconditioning

The underlying mechanism of chemical/pharmacological preconditioning and their neuroprotective potential as strategies for clinical prevention and treatment of cerebral ischemia are gradually being explored (68–72). Isoflurane, halothane, and other inhalational anesthetics could lead to a subsequent protective phenotype against subsequent ischemic injury via promoting the antagonism of NMDA and AMPA receptors (68, 73). For example, adult male rats with 2% isoflurane inhalation preconditioning for half an hour showed smaller brain infarct sizes after focal ischemia (79). It is considered that sevoflurane possesses smaller infarct sizes and better motor coordination after ischemia (73). Halothane has no clinical feasibility due to the likelihood of hepatotoxicity and other systemic side effects although it could be potentially neuroprotective (73). Using combined inhaled anesthetics might be an alternative for extended neuroprotection (80). Low dose of lipopolysaccharide (LPS) preconditioning can later impart ischemic tolerance in the brain in rats (81, 82). Exposure to LPS for 4 consecutive days could significantly reduce neuronal death in mice (74). Interestingly, although LPS functioned by suppressing TNF-α signaling, TNF-α is also a necessary prerequisite for LPS-mediated neuroprotection, predicting the dual roles of TNF-α in LPS-mediated neuroprotection (75–77, 81, 83). Exogenous agents that protect the tricarboxylic acid cycle energy metabolism pathway can effectively induce neuroprotection. The pretreatment of chemical reagents to inhibit oxidative phosphorylation of neurons in the CA1 region of the hippocampus slices in rats can significantly reduce the oxidative stress damage mediated by a large amount of oxygen free radicals generated after hypoxia (84). Transient ischemic stimulation resulted in the release of adenosine and activates the ATP-sensitive K^+^ channels in the brain to mediate neuroprotection, and pharmacological preconditioning with adenosine receptor agonist can mimic the neuroprotective effects (85). 5-methoxyindole-2-carboxylic acid preconditioning contributed to the neuroprotective effects through regulating the Nrf2 signaling, which decreased dihydrolipoamide dehydrogenase activity and increased nicotinamide adenine dinucleotide: ubiquinone oxidoreductase-1 expression (86). Research results indicated that 3-nitropropionic acid preconditioning mediated obvious neuroprotection after cerebral ischemia in rats (87). However, nitrous oxide preconditioning had little or no neuroprotective effect in focal or global ischemia models, it may even repress the neuroprotective effects when used in conjugation with other inhalational anesthetics (73). Jackson et al. (88) preconditioned rats with daily metformin treatments for 2 weeks before transient forebrain global ischemia. They found that metformin preconditioning increased mitochondrial biogenesis and reduced apoptotic cell death (88). Research showed that dexmedetomidine preconditioning could protect against global cerebral ischemic injury following cardiac arrest and was associated with increased HIF-1α and VEGF expression (89). Preconditioning with recombinant high-mobility group box 1 (rHMGB1) can protect the brain against ischemic damage, which is associated with activated TLR4/interleukin-1R-associated kinase-M signaling in microglia. The lipid kinase sphingosine kinase 2 (SPK-2), an important mediator of ischemic tolerance induced by isoflurane and hypoxia preconditioning (90), can upregulate sphingosine-1-phosphate that promoted the expression of chemokine (C–C motif) ligand 2 to mediated tolerance (91). Several *in vivo* studies have shown that metformin and estrogen involve in preconditioning neuroprotection (251).

#### Other Methods of Preconditioning

Continuous electroacupuncture pretreatment can reduce infarct size and improve motor function after ischemia in mice (252). CSD preconditioning of rat brain can resist I/R damage and elicit improved prognosis by activating AMPK-mediated autophagy (38). A 3-week preconditioning period with a ketogenic diet elevated extracellular adenosine levels, improved rCBF, and increased HIFs and HIF-regulated genes in mice with MCAO (253). Other ischemic tolerance-inducing stimuli include exercise and transcranial low-level light therapy (254, 255).

## Clinical Uses and Concerns

IPreC strategies involve the application of non-lethal but noxious stressors or stimuli, but an inevitable problem is that preconditioning stimuli also have the potential to cause fatal damage. Therefore, preconditioning strategies applied to clinical patients must ensure their security and effectiveness. In spite of its apparent popularity and the largely flourishing trials in the experimental and clinical scenario, the next challenge of research in the area of ischemic neuroprotection is to find the most effective IPreC method, concerning the time window and proper dosage for clinical application.

At present, the results of studies on the protective effect of RIPreC in cerebral ischemia are not consistent: Some studies have shown the effectiveness of LIPreC, and some results are contrary to the above results (27, 256, 257). Limb-RIPreC is seen as a more promising neuroprotective strategy in terms of better tolerance and improved safety of the brain to I/R injury (218, 258, 259), and limb-RIPreC might be the future choice of clinical ischemic adjuvant treatment. For example, subarachnoid hemorrhage (SAH) can lead to delayed cerebral ischemia due to vasospasm, which is considered to be a suitable clinical situation for RIPreC (260). It is reported that the glycerol level and the ratio of lactic acid:pyruvic acid all decreased in SAH patients receiving limb-RIPreC, and these neuroprotective effects lasted up to 2 days (28, 261). Recently, it was found that, in patients with symptomatic atherosclerotic intracranial arterial stenosis, bilateral arm IPreC could reduce the occurrence of stroke (218). Cross-preconditioning provides new ideas for clinical ischemia treatment, especially chemical/pharmacological pretreatment strategies. For example, inhaled anesthetics can effectively mediate neuroprotection and have been already used in surgical procedures (262). Further experimental or clinical research on the mechanism of IPreC-induced cerebral ischemic tolerance are especially necessary.

## Conclusion

Proper stimulus can trigger ischemic tolerance; however, it should be noted that there is no clear line between preconditioning stimuli and lethal stimuli. For safe and regular development of ischemic tolerance, various mutually cross-linked factors that influence the deleterious effect of IPreC need special attention, for example, the endogenous signaling pathways for neuronal survival, the exact window of protection after the final insult, the duration of tolerance, the safety margin, and possible side effects in patients. Every IPreC stumili has its advantages as well as limitations, and choosing the most effective preconditioning inducer for clinical use is not always easy. Extensive studies with different combinations of stimulus should be conducted, especially cross-preconditioning, and the strategy of RIPreC combined with chemical or pharmacological pretreatment might be the future trend in clinical practice as they seem to hold more promise for combating stroke and related neurological disorders.

## Author Contributions

MX and YH: designing and writing original draft. LF, XiW, and XuW: drawing. DM and JF: writing review and editing. All authors contributed to manuscript revision, read, and approved the submitted version.

## Conflict of Interest

The authors declare that the research was conducted in the absence of any commercial or financial relationships that could be construed as a potential conflict of interest.

## References

[1] Virani SS Alonso A Benjamin EJ Bittencourt MS Callaway CW Carson AP. Epidemiology American Heart Association Council on, Committee Prevention Statistics, and Subcommittee Stroke Statistics, Heart Disease and Stroke Statistics-2020 update: a report from the American Heart Association. Circulation. (2020) 141:e139–596. 10.1161/CIR.0000000000000746 31992061

[2] Donnan GA Davis SM. Stroke: expanded indications for stroke thrombolysis–what next? Nat Rev Neurol. (2012) 8:482–3. 10.1038/nrneurol.2012.151 22847384

[3] Schmidt-Kastner R. Genomic approach to selective vulnerability of the hippocampus in brain ischemia-hypoxia. Neuroscience. (2015) 309:259–79. 10.1016/j.neuroscience.2015.08.034 26383255

[4] Albers GW Bates VE Clark WM Bell R Verro P Hamilton SA. Intravenous tissue-type plasminogen activator for treatment of acute stroke: the Standard Treatment with Alteplase to Reverse Stroke (STARS) study. JAMA. (2000) 283:1145–50. 10.1001/jama.283.9.1145 10703776

[5] Kitagawa K. Ischemic tolerance in the brain: endogenous adaptive machinery against ischemic stress. J Neurosci Res. (2012) 90:1043–54. 10.1002/jnr.23005 22302606

[6] Dirnagl U Simon RP Hallenbeck JM. Ischemic tolerance and endogenous neuroprotection. Trends Neurosci. (2003) 26:248–54. 10.1016/S0166-2236(03)00071-7 12744841

[7] Stapels M Piper C Yang T Li M Stowell C Xiong ZG. Polycomb group proteins as epigenetic mediators of neuroprotection in ischemic tolerance. Sci Signal. (2010) 3:ra15. 10.1126/scisignal.2000502 20197544 PMC3878609

[8] Dirnagl U Becker K Meisel A. Preconditioning and tolerance against cerebral ischaemia: from experimental strategies to clinical use. Lancet. (2009) 8:398–412. 10.1016/S1474-4422(09)70054-7 19296922 PMC2668955

[9] Jin Z Wu J Yan LJ. Chemical conditioning as an approach to ischemic stroke tolerance: mitochondria as the target. Int J Mol Sci. (2016) 17:351. 10.3390/ijms17030351 27005615 PMC4813212

[10] Wiegand F Liao W Busch C Castell S Knapp F Lindauer U. Respiratory chain inhibition induces tolerance to focal cerebral ischemia. J Cereb Blood Flow Metab. (1999) 19:1229–37. 10.1097/00004647-199911000-00007 10566969

[11] Bordet R Deplanque D Maboudou P Puisieux F Pu Q Robin E. Increase in endogenous brain superoxide dismutase as a potential mechanism of lipopolysaccharide-induced brain ischemic tolerance. J Cereb Blood Flow Metab. (2000) 20:1190–6. 10.1097/00004647-200008000-00004 10950379

[12] Yanamoto H Xue JH Miyamoto S Nagata I Nakano Y Murao K. Spreading depression induces long-lasting brain protection against infarcted lesion development via BDNF gene-dependent mechanism. Brain Res. (2004) 1019:178–88. 10.1016/j.brainres.2004.05.105 15306252

[13] Marber MS Latchman DS Walker JM Yellon DM Cardiac stress protein elevation 24 hours after brief ischemia or heat stress is associated with resistance to myocardial infarction. Circulation. (1993) 88:1264–72. 10.1161/01.CIR.88.3.1264 8353888

[14] Murry CE Jennings RB Reimer KA. Preconditioning with ischemia: a delay of lethal cell injury in ischemic myocardium. Circulation. (1986) 74:1124–36. 10.1161/01.CIR.74.5.1124 3769170

[15] Przyklenk K Bauer B Ovize M Kloner RA Whittaker P. Regional ischemic 'preconditioning' protects remote virgin myocardium from subsequent sustained coronary occlusion. Circulation. (1993) 87:893–9. 10.1161/01.CIR.87.3.893 7680290

[16] Hausenloy DJ Yellon DM. Remote ischaemic preconditioning: underlying mechanisms and clinical application. Cardiovas Res. (2008) 79:377–86. 10.1093/cvr/cvn114 18456674

[17] Sandhu R Diaz RJ Mao GD Wilson GJ. Ischemic preconditioning: differences in protection and susceptibility to blockade with single-cycle versus multicycle transient ischemia. Circulation. (1997) 96:984–95. 10.1161/01.CIR.96.3.984 9264510

[18] Chen C Sun L Zhang W Tang Y Li X Jing R. Limb ischemic preconditioning ameliorates renal microcirculation through activation of PI3K/Akt/eNOS signaling pathway after acute kidney injury. Eur J Med Res. (2020) 25:10. 10.1186/s40001-020-00407-4 32192513 PMC7081586

[19] Cho YJ Kim WH. Perioperative cardioprotection by remote ischemic conditioning. Int J Mol Sci. (2019) 20:4839. 10.3390/ijms20194839 31569468 PMC6801656

[20] Schurr A Reid KH Tseng MT West C Rigor BM. Adaptation of adult brain tissue to anoxia and hypoxia *in vitro*. Brain Res. (1986) 374:244-8. 10.1016/0006-8993(86)90418-X 3719335

[21] Chopp M Chen H Ho KL Dereski MO Brown E Hetzel FW. Transient hyperthermia protects against subsequent forebrain ischemic cell damage in the rat. Neurology. (1989) 39:1396–8. 10.1212/WNL.39.10.1396 2797463

[22] Kitagawa K Matsumoto M Tagaya M Hata R Ueda H Niinobe M. 'Ischemic tolerance' phenomenon found in the brain. Brain Res. (1990) 528:21-4. 10.1016/0006-8993(90)90189-I 2245337

[23] Kirino T Tsujita Y Tamura A. Induced tolerance to ischemia in gerbil hippocampal neurons. J Cereb Blood Flow Metab. (1991) 11:299–307. 10.1038/jcbfm.1991.62 1997501

[24] Chen J Graham SH Zhu RL Simon RP. Stress proteins and tolerance to focal cerebral ischemia. J Cereb Blood Flow Metab. (1996) 16:566–77. 10.1097/00004647-199607000-00006 8964795

[25] Glazier SS O'Rourke DM Graham DI Welsh FA. Induction of ischemic tolerance following brief focal ischemia in rat brain. J Cereb Blood Flow Metab. (1994) 14:545–53. 10.1038/jcbfm.1994.68 8014201

[26] Stetler RA Leak RK Gan Y Li P Zhang F Hu X. Preconditioning provides neuroprotection in models of CNS disease: paradigms and clinical significance. Progr Neurobiol. (2014) 114:58–83. 10.1016/j.pneurobio.2013.11.005 24389580 PMC3937258

[27] Healy DA Boyle E McCartan D Bourke M Medani M Ferguson J. A multicenter pilot randomized controlled trial of remote ischemic preconditioning in major vascular surgery. Vascul Endovasc Surg. (2015) 49:220–7. 10.1177/1538574415614404 26574485

[28] Koch S Katsnelson M Dong C Perez-Pinzon M. Remote ischemic limb preconditioning after subarachnoid hemorrhage: a phase Ib study of safety and feasibility. Stroke. (2011) 42:1387–91. 10.1161/STROKEAHA.110.605840 21415404 PMC3082628

[29] Sharp FR Ran R Lu A Tang Y Strauss KI Glass T. Hypoxic preconditioning protects against ischemic brain injury. NeuroRx. (2004) 1:26–35. 10.1602/neurorx.1.1.26 15717005 PMC534910

[30] Yang J Liu C Du X Liu M Ji X Du H. Hypoxia inducible factor 1α plays a key role in remote ischemic preconditioning against stroke by modulating inflammatory responses in rats. J Am Heart Assoc. (2018) 7: e007589. 10.1161/JAHA.117.007589 29478025 PMC5866324

[31] Gu GJ Li YP Peng ZY Xu JJ Kang ZM Xu WG. Mechanism of ischemic tolerance induced by hyperbaric oxygen preconditioning involves upregulation of hypoxia-inducible factor-1alpha and erythropoietin in rats. J Appl Physiol. (2008) 104:1185–91. 10.1152/japplphysiol.00323.2007 18174394

[32] Yenari MA Han HS. Neuroprotective mechanisms of hypothermia in brain ischaemia. Nat Rev. Neurosci. (2012) 13:267–78. 10.1038/nrn3174 22353781

[33] Li H Yin J Li L Deng J Feng C Zuo Z. Isoflurane postconditioning reduces ischemia-induced nuclear factor-κB activation and interleukin 1β production to provide neuroprotection in rats and mice. Neurobiol Dis. (2013) 54:216–24. 10.1016/j.nbd.2012.12.014 23313315 PMC3628970

[34] Lee JJ Li L Jung HH Zuo Z. Postconditioning with isoflurane reduced ischemia-induced brain injury in rats. Anesthesiology. (2008) 108:1055–62. 10.1097/ALN.0b013e3181730257 18497606 PMC2666347

[35] Della-Morte D Dave KR DeFazio RA Bao YC Raval AP Perez-Pinzon MA. Resveratrol pretreatment protects rat brain from cerebral ischemic damage via a sirtuin 1-uncoupling protein 2 pathway. Neuroscience. (2009) 159:993–1002. 10.1016/j.neuroscience.2009.01.017 19356683 PMC2668125

[36] Li Z Fang F Wang Y Wang L. Resveratrol protects CA1 neurons against focal cerebral ischemic reperfusion-induced damage via the ERK-CREB signaling pathway in rats. Pharmacol Biochem Behav. (2016) 21–7. 10.1016/j.pbb.2016.04.007 27143440

[37] Wu X Qian Z Ke Y Du F Zhu L. Ginkgolide B preconditioning protects neurons against ischaemia-induced apoptosis. J Cell Mol Med. (2009) 13:4474–83. 10.1111/j.1582-4934.2008.00551.x 19602048 PMC4515063

[38] Shen P Hou S Zhu M Zhao M Ouyang Y Feng J. Cortical spreading depression preconditioning mediates neuroprotection against ischemic stroke by inducing AMP-activated protein kinase-dependent autophagy in a rat cerebral ischemic/reperfusion injury model. J Neurochem. (2017) 140:799–813. 10.1111/jnc.13922 27987215

[39] Shen PP Hou S Ma D Zhao MM Zhu MQ Zhang JD. Cortical spreading depression-induced preconditioning in the brain. Neural Regenerat Res. (2016) 11:1857–64. 10.4103/1673-5374.194759 28123433 PMC5204245

[40] Jin Z Liang J Wang J Kolattukudy PE. Delayed brain ischemia tolerance induced by electroacupuncture pretreatment is mediated via MCP-induced protein 1. J Neuroinflamm. (2013) 10:63. 10.1186/1742-2094-10-63 23663236 PMC3701471

[41] Chen HI Hsieh SY Yang FL Hsu YH Lin CC. Exercise training attenuates septic responses in conscious rats. Med Sci Sports Exerc. (2007) 39:435–42. 10.1249/mss.0b013e31802d11c8 17473769

[42] Traystman RJ. Animal models of focal and global cerebral ischemia. ILAR J. (2003) 44:85–95. 10.1093/ilar.44.2.85 12652003

[43] Bahjat FR Gesuete R Stenzel-Poore MP. Steps to translate preconditioning from basic research to the clinic. Transl Stroke Res. (2013) 4:89–103. 10.1007/s12975-012-0223-4 23504609 PMC3595131

[44] Wu C Zhan RZ Qi S Fujihara H Taga K Shimoji K. A forebrain ischemic preconditioning model established in C57Black/Crj6 mice. J Neurosci Methods. (2001) 107:101–6. 10.1016/S0165-0270(01)00356-9 11389947

[45] Zhang QG Han D Xu J Lv Q Wang R Yin XH. Ischemic preconditioning negatively regulates plenty of SH3s-mixed lineage kinase 3-Rac1 complex and c-Jun N-terminal kinase 3 signaling via activation of Akt. Neuroscience. (2006) 143:431–44. 10.1016/j.neuroscience.2006.07.049 16973299

[46] Liu C Chen S Kamme F Hu BR. Ischemic preconditioning prevents protein aggregation after transient cerebral ischemia. Neuroscience. (2005) 134:69–80. 10.1016/j.neuroscience.2005.03.036 15939539 PMC3518067

[47] Geddes JW Pettigrew LC Holtz ML Craddock SD Maines MD. Permanent focal and transient global cerebral ischemia increase glial and neuronal expression of heme oxygenase-1, but not heme oxygenase-2, protein in rat brain. Neuroscience letters. (1996) 210:205–8. 10.1016/0304-3940(96)12703-8 8805131

[48] Kuzuya T Hoshida S Yamashita N Fuji H Oe H Hori M. Delayed effects of sublethal ischemia on the acquisition of tolerance to ischemia. Circ Res. (1993) 72:1293–9. 10.1161/01.RES.72.6.1293 8495557

[49] Miura T Adachi T Ogawa T Iwamoto T Tsuchida A Iimur O. Myocardial infarct size-Limiting effect of ischemic preconditioning: its natural decay and the effect of repetitive preconditioning. Cardiovasc Pathol. (1992) 1:147–54. 10.1016/1054-8807(92)90018-J 25990126

[50] Hu S Dong HL Li YZ Luo ZJ Sun L Yang QZ. Effects of remote ischemic preconditioning on biochemical markers and neurologic outcomes in patients undergoing elective cervical decompression surgery: a prospective randomized controlled trial. J Neurosurg Anesthesiol. (2010) 22:46–52. 10.1097/ANA.0b013e3181c572bd 19996767

[51] Singh D Chopra K. Evidence of the role of angiotensin AT(1) receptors in remote renal preconditioning of myocardium. Methods Find Exp Clin Pharmacol. (2004) 26:117–22. 10.1358/mf.2004.26.2.800064 15071610

[52] Liu M Liang Y Chigurupati S Lathia JD Pletnikov M Sun Z. Acute kidney injury leads to inflammation and functional changes in the brain. J Am Soc Nephrol. (2008) 19:1360–70. 10.1681/ASN.2007080901 18385426 PMC2440297

[53] Rehni AK Shri R Singh M. Remote ischaemic preconditioning and prevention of cerebral injury. Indian J Exp Biol. (2007) 45:247–52. 17373368

[54] Varga S Juhász L Gál P Bogáts G Boros M Palásthy Z. Neuronal nitric oxide mediates the anti-inflammatory effects of intestinal ischemic preconditioning. J Surg Res. (2019) 244:241–50. 10.1016/j.jss.2019.06.053 31301480

[55] Ateş E Genç E Erkasap N Erkasap S Akman S Firat P. Renal protection by brief liver ischemia in rats. Transplantation. (2002) 74:1247–51. 10.1097/00007890-200211150-00009 12451261

[56] Yang G Yang Y Li Y Hu Z. Remote liver ischaemic preconditioning protects rat brain against cerebral ischaemia-reperfusion injury by activation of an AKT-dependent pathway. Exp Physiol. (2020) 105:852–63. 10.1113/EP088394 32134522

[57] Okada M Nakanishi H Tamura A Urae A Mine K Yamamoto K. Long-term spatial cognitive impairment after middle cerebral artery occlusion in rats: no involvement of the hippocampus. J Cereb Blood Flow Metab. (1995) 15:1012–21. 10.1038/jcbfm.1995.127 7593333

[58] Li K Zhou H Zhan L Shi Z Sun W Liu D. Hypoxic preconditioning maintains GLT-1 against transient global cerebral ischemia through upregulating Cx43 and inhibiting c-Src. Front Mol Neurosci. (2018) 11:344. 10.3389/fnmol.2018.00344 30323740 PMC6172853

[59] Bigdeli MR. Neuroprotection caused by hyperoxia preconditioning in animal stroke models. Sci World J. (2011) 11:403–21. 10.1100/tsw.2011.23 21336456 PMC5719998

[60] Cheng O Ostrowski RP Wu B Liu W Chen C Zhang JH. Cyclooxygenase-2 mediates hyperbaric oxygen preconditioning in the rat model of transient global cerebral ischemia. Stroke. (2011) 42:484–90. 10.1161/STROKEAHA.110.604421 21164135 PMC3026922

[61] Ostrowski RP Graupner G Titova E Zhang J Chiu J Dach N. The hyperbaric oxygen preconditioning-induced brain protection is mediated by a reduction of early apoptosis after transient global cerebral ischemia. Neurobiol Dis. (2008) 29:1–13. 10.1016/j.nbd.2007.07.020 17822911 PMC2190110

[62] Selvaraj UM Ortega SB Hu R Gilchrist R Kong X Partin A. Preconditioning-induced CXCL12 upregulation minimizes leukocyte infiltration after stroke in ischemia-tolerant mice. J Cereb Blood Flow Metabol. (2017) 37:801–13. 10.1177/0271678X16639327 27006446 PMC5363460

[63] Hemmen TM Raman R Guluma KZ Meyer BC Gomes JA Cruz-Flores S. Intravenous thrombolysis plus hypothermia for acute treatment of ischemic stroke (ICTuS-L): final results. Stroke. (2010) 41:2265–70. 10.1161/STROKEAHA.110.592295 20724711 PMC2947593

[64] Yunoki M Nishio S Ukita N Anzivino MJ Lee KS. Hypothermic preconditioning induces rapid tolerance to focal ischemic injury in the rat. Exp Neurol. (2003) 181:291–300. 10.1016/S0014-4886(03)00056-6 12782001

[65] Nishio S Yunoki M Chen ZF Anzivino MJ Lee KS. Ischemic tolerance in the rat neocortex following hypothermic preconditioning. J Neurosurg. (2000) 93:845–51. 10.3171/jns.2000.93.5.0845 11059667

[66] Ikeda T Xia XY Xia YX Ikenoue T. Hyperthermic preconditioning prevents blood-brain barrier disruption produced by hypoxia-ischemia in newborn rat. Brain Res. (1999) 117:53–8. 10.1016/S0165-3806(99)00097-8 10536232

[67] Du F Zhu L Qian ZM Wu XM Yung WH Ke Y. Hyperthermic preconditioning protects astrocytes from ischemia/reperfusion injury by up-regulation of HIF-1 alpha expression and binding activity. Biochim Biophys Acta. (2010) 1802:1048–53. 10.1016/j.bbadis.2010.06.013 20599612

[68] Wang L Traystman RJ Murphy SJ. Inhalational anesthetics as preconditioning agents in ischemic brain. Curr Opin Pharmacol. (2008) 8:104–10. 10.1016/j.coph.2007.09.005 17962069 PMC2254937

[69] Zhang HP Sun YY Chen XM Yuan LB Su BX Ma R The neuroprotective effects of isoflurane preconditioning in a murine transient global cerebral ischemia-reperfusion model: the role of the Notch signaling pathway. Neuromol Med. (2014) 16:191–204. 10.1007/s12017-013-8273-7 24197755

[70] Wang H Lu S Yu Q Liang W Gao H Li P. Sevoflurane preconditioning confers neuroprotection via anti-inflammatory effects. Front Biosci. (2011) 3:604–15. 10.2741/e273 21196338

[71] Li L Zuo Z. Isoflurane preconditioning improves short-term and long-term neurological outcome after focal brain ischemia in adult rats. Neuroscience. (2009) 164:497–506. 10.1016/j.neuroscience.2009.08.011 19679170 PMC2762026

[72] Bantel C Maze M Trapp S. Neuronal preconditioning by inhalational anesthetics: evidence for the role of plasmalemmal adenosine triphosphate-sensitive potassium channels. Anesthesiology. (2009) 110:986–95. 10.1097/ALN.0b013e31819dadc7 19352153 PMC2930813

[73] Kitano H Kirsch JR Hurn PD Murphy SJ Inhalational anesthetics as neuroprotectants or chemical preconditioning agents in ischemic brain. J Cereb Blood Flow Metab. (2007) 27:1108–28. 10.1038/sj.jcbfm.9600410 17047683 PMC2266688

[74] Chen Z Jalabi W Shpargel KB Farabaugh KT Dutta R Yin X. Lipopolysaccharide-induced microglial activation and neuroprotection against experimental brain injury is independent of hematogenous TLR4. J Neurosci. (2012) 32:11706–15. 10.1523/JNEUROSCI.0730-12.2012 22915113 PMC4461442

[75] Rosenzweig HL Minami M Lessov NS Coste SC Stevens SL Henshall DC. Endotoxin preconditioning protects against the cytotoxic effects of TNFalpha after stroke: a novel role for TNFalpha in LPS-ischemic tolerance. J Cereb Blood Flow Metab. (2007) 27:1663–74. 10.1038/sj.jcbfm.9600464 17327883

[76] Sakata H Narasimhan P Niizuma K Maier CM Wakai T Chan PH. Interleukin 6-preconditioned neural stem cells reduce ischaemic injury in stroke mice. Brain. (2012) 135:3298–310. 10.1093/brain/aws259 23169920 PMC3501976

[77] Ohtsuki T Ruetzler CA Tasaki K Hallenbeck JM. Interleukin-1 mediates induction of tolerance to global ischemia in gerbil hippocampal CA1 neurons. J Cereb Blood Flow Metab. (1996) 16:1137–42. 10.1097/00004647-199611000-00007 8898685

[78] Jensen HA Loukogeorgakis S Yannopoulos F Rimpiläinen E Petzold A Tuominen H. Remote ischemic preconditioning protects the brain against injury after hypothermic circulatory arrest. Circulation. (2011) 123:714–21. 10.1161/CIRCULATIONAHA.110.986497 21300953

[79] Zheng S Zuo Z. Isoflurane preconditioning induces neuroprotection against ischemia via activation of P38 mitogen-activated protein kinases. Mol Pharmacol. (2004) 65:1172–80. 10.1124/mol.65.5.1172 15102945

[80] Nunes RR Duval Neto GF de Alencar JC Franco SB de Andrade NQ Dumaresq DM Anesthetics, cerebral protection and preconditioning. Brazil J Anesthesiol. (2013) 63:119–28. 10.1016/S0034-7094(13)70204-6 24565096

[81] Vartanian KB Stevens SL Marsh BJ Williams-Karnesky R Lessov NS Stenzel-Poore MP. LPS preconditioning redirects TLR signaling following stroke: TRIF-IRF3 plays a seminal role in mediating tolerance to ischemic injury. J Neuroinflamm. (2011) 8:140. 10.1186/1742-2094-8-140 21999375 PMC3217906

[82] Lin HY Wu CL Huang CC. The Akt-endothelial nitric oxide synthase pathway in lipopolysaccharide preconditioning-induced hypoxic-ischemic tolerance in the neonatal rat brain. Stroke. (2010) 41:1543–51. 10.1161/STROKEAHA.109.574004 20508195

[83] Spera PA Ellison JA Feuerstein GZ Barone FC. IL-10 reduces rat brain injury following focal stroke. Neurosci Lett. (1998) 251:189–92. 10.1016/S0304-3940(98)00537-0 9726375

[84] Riepe MW Esclaire F Kasischke K Schreiber S Nakase H Kempski O. Increased hypoxic tolerance by chemical inhibition of oxidative phosphorylation: “chemical preconditioning”. J Cereb Blood Flow Metab. (1997) 17:257–64. 10.1097/00004647-199703000-00002 9119898

[85] Heurteaux C Lauritzen I Widmann C Lazdunski M. Essential role of adenosine, adenosine A1 receptors, and ATP-sensitive K+ channels in cerebral ischemic preconditioning. Proc Natl Acad Sci USA. (1995) 92:4666–70. 10.1073/pnas.92.10.4666 7753861 PMC42005

[86] Wu J Li R Li W Ren M Thangthaeng N Sumien N. Administration of 5-methoxyindole-2-carboxylic acid that potentially targets mitochondrial dihydrolipoamide dehydrogenase confers cerebral preconditioning against ischemic stroke injury. Free Rad Biol Med. (2017) 113:244–54. 10.1016/j.freeradbiomed.2017.10.008 29017857 PMC5699942

[87] Zhu H Sun S Li H Xu Y. Cerebral ischemic tolerance induced by 3-nitropropionic acid is associated with increased expression of erythropoietin in rats. J Huazhong Univers Sci Technol. (2006) 26:440–3. 10.1007/s11596-006-0416-8 17120743

[88] Jackson CW Escobar I Xu J Perez-Pinzon MA. Effects of ischemic preconditioning on mitochondrial and metabolic neruoprotection: 5′ adenosine monophosphate-activated protein kinase and sirtuins. Brain Circ. (2018) 4:54–61. 10.4103/bc.bc_7_18 30276337 PMC6126241

[89] Ding XD Zheng NN Cao YY Zhao GY Zhao P. Dexmedetomidine preconditioning attenuates global cerebral ischemic injury following asphyxial cardiac arrest. Int J Neurosci. (2016) 126:249–56. 10.3109/00207454.2015.1005291 25565380

[90] Yung LM Wei Y Qin T Wang Y Smith CD Waeber C. Sphingosine kinase 2 mediates cerebral preconditioning and protects the mouse brain against ischemic injury. Stroke. (2012) 43:199–204. 10.1161/STROKEAHA.111.626911 21980199 PMC3246529

[91] Wacker BK Perfater JL Gidday JM. Hypoxic preconditioning induces stroke tolerance in mice via a cascading HIF, sphingosine kinase, and CCL2 signaling pathway. J Neurochem. (2012) 123:954–62. 10.1111/jnc.12047 23043544 PMC3514614

[92] Felgueiras R Magalhães R Silva MR Silva MC Correia M. Transient ischemic attack: incidence and early risk of stroke in northern Portugal from 1998-2000 to 2009-2011. Int J Stroke. (2019) 15:278–88. 10.1177/1747493019830322 30734663

[93] Wang WW Chen DZ Zhao M Yang XF Gong DR. Prior transient ischemic attacks may have a neuroprotective effect in patients with ischemic stroke. Archiv Med Sci. (2017) 13:1057–61. 10.5114/aoms.2016.63744 28883846 PMC5575216

[94] Liu Y Zhu S Wang Y Hu J Xu L Ding L. Neuroprotective effect of ischemic preconditioning in focal cerebral infarction: relationship with upregulation of vascular endothelial growth factor. Neural Regenerat Res. (2014) 9:1117–21. 10.4103/1673-5374.135313 25206770 PMC4146099

[95] Zsuga J Gesztelyi R Juhasz B Kemeny-Beke A Fekete I Csiba L. Prior transient ischemic attack is independently associated with lesser in-hospital case fatality in acute stroke. Psychiatry Clin Neurosci. (2008) 62:705–12. 10.1111/j.1440-1819.2008.01874.x 19068008

[96] Lovett JK Dennis MS Sandercock PA Bamford J Warlow CP Rothwell PM. Very early risk of stroke after a first transient ischemic attack. Stroke. (2003) 34:e138–40. 10.1161/01.STR.0000080935.01264.91 12855835

[97] Weih M Kallenberg K Bergk A Dirnagl U Harms L Wernecke KD. Attenuated stroke severity after prodromal TIA: a role for ischemic tolerance in the brain? Stroke. (1999) 30:1851–4. 10.1161/01.STR.30.9.1851 10471435

[98] Béjot Y Aboa-Eboulé C Marie C Giroud M. [Neuroprotective effect of transient ischemic attack]. Presse Med. (2011) 40:167–72. 10.1016/j.lpm.2010.09.024 21112179

[99] Wegener S Gottschalk B Jovanovic V Knab R Fiebach JB Schellinger PD Transient ischemic attacks before ischemic stroke: preconditioning the human brain? A multicenter magnetic resonance imaging study. Stroke. (2004) 35:616–21. 10.1161/01.STR.0000115767.17923.6A 14963288

[100] Sitzer M Foerch C Neumann-Haefelin T Steinmetz H Misselwitz B Kugler C. Transient ischaemic attack preceding anterior circulation infarction is independently associated with favourable outcome. J Neurol Neurosurg Psychiatry. (2004) 75:659–60. 10.1136/jnnp.2003.015875 15026523 PMC1739003

[101] Schaller B. Ischemic preconditioning as induction of ischemic tolerance after transient ischemic attacks in human brain: its clinical relevance. Neurosci Lett. (2005) 377:206-11. 10.1016/j.neulet.2004.12.004 15755527

[102] Johnston SC. Ischemic preconditioning from transient ischemic attacks? Data from the Northern California TIA Study. Stroke. (2004) 35:2680–2. 10.1161/01.STR.0000143322.20491.0f 15388902

[103] Lavine SD Masri LS Levy ML Giannotta SL. Temporary occlusion of the middle cerebral artery in intracranial aneurysm surgery: time limitation and advantage of brain protection. J Neurosurg. (1997) 87:817–24. 10.3171/jns.1997.87.6.0817 9384389

[104] Durukan A Tatlisumak T. Preconditioning-induced ischemic tolerance: a window into endogenous gearing for cerebroprotection. Exp Transl Stroke Med. (2010) 2:2. 10.1186/2040-7378-2-2 20298534 PMC2830184

[105] Gidday JM. Cerebral preconditioning and ischaemic tolerance. Nat Rev Neurosci. (2006) 7:437–48. 10.1038/nrn1927 16715053

[106] Yang T Sun Y Mao L Zhang M Li Q Zhang L. Brain ischemic preconditioning protects against ischemic injury and preserves the blood-brain barrier via oxidative signaling and Nrf2 activation. Redox biology. (2018) 17:323–337. 10.1016/j.redox.2018.05.001 29775963 PMC6007054

[107] Zhang N Gao G Bu X Han S Fang L Li J. Neuron-specific phosphorylation of c-Jun N-terminal kinase increased in the brain of hypoxic preconditioned mice. Neuroscience letters. (2007) 423:219–24. 10.1016/j.neulet.2007.07.028 17709198

[108] Corbett D Nurse S. The problem of assessing effective neuroprotection in experimental cerebral ischemia. Progr Neurobiol. (1998) 54:531–48. 10.1016/S0301-0082(97)00078-6 9550190

[109] Masada T Hua Y Xi G Ennis SR Keep RF. Attenuation of ischemic brain edema and cerebrovascular injury after ischemic preconditioning in the rat. J Cereb Blood Flow Metab. (2001) 21:22–33. 10.1097/00004647-200101000-00004 11149665

[110] Miyashita K Abe H Nakajima T Ishikawa A Nishiura M Sawada T. Induction of ischaemic tolerance in gerbil hippocampus by pretreatment with focal ischaemia. Neuroreport. (1994) 6:46–8. 10.1097/00001756-199412300-00013 7703426

[111] Peart JN Headrick JP. Sustained cardioprotection: exploring unconventional modalities. Vascul Pharmacol. (2008) 49:63–70. 10.1016/j.vph.2008.07.001 18675381

[112] Schulz R Post H Vahlhaus C Heusch G. Ischemic preconditioning in pigs: a graded phenomenon: its relation to adenosine and bradykinin. Circulation. (1998) 98:1022–9. 10.1161/01.CIR.98.10.1022 9737523

[113] Jennings RB Sebbag L Schwartz LM Crago MS Reimer KA. Metabolism of preconditioned myocardium: effect of loss and reinstatement of cardioprotection. J Mol Cell Cardiol. (2001) 33:1571–88. 10.1006/jmcc.2001.1425 11549338

[114] Lin CH Chen PS Gean PW. Glutamate preconditioning prevents neuronal death induced by combined oxygen-glucose deprivation in cultured cortical neurons. Eur J Pharmacol. (2008) 589:85–93. 10.1016/j.ejphar.2008.05.047 18589412

[115] Grabb MC Choi DW. Ischemic tolerance in murine cortical cell culture: critical role for NMDA receptors. J Neurosci. (1999) 19:1657–62. 10.1523/JNEUROSCI.19-05-01657.1999 10024352 PMC6782179

[116] Guo ZH Li F Wang WZ. The mechanisms of brain ischemic insult and potential protective interventions. Neurosci Bull. (2009) 25:139–52. 10.1007/s12264-009-0104-3 19448688 PMC5552559

[117] Bhuiyan MI Kim YJ. Mechanisms and prospects of ischemic tolerance induced by cerebral preconditioning. Int Neurourol J. (2010) 14:203–12. 10.5213/inj.2010.14.4.203 21253330 PMC3021810

[118] Benardete EA Bergold PJ. Genomic analysis of ischemic preconditioning in adult rat hippocampal slice cultures. Brain Res. (2009) 1292:107–22. 10.1016/j.brainres.2009.07.027 19631194

[119] Hawaleshka A Jacobsohn E. Ischaemic preconditioning: mechanisms and potential clinical applications. Can J Anaesthesia. (1998) 45:670–82. 10.1007/BF03012100 9717602

[120] Steiger HJ Hänggi D. Ischaemic preconditioning of the brain, mechanisms and applications. Acta Neurochirurgica. (2007) 149:1–10. 10.1007/s00701-006-1057-1 17151832

[121] Yoshida M Nakakimura K Cui YJ Matsumoto M Sakabe T. Adenosine A(1) receptor antagonist and mitochondrial ATP-sensitive potassium channel blocker attenuate the tolerance to focal cerebral ischemia in rats. J Cereb Blood Flow Metab. (2004) 24:771–9. 10.1097/01.WCB.0000122742.72175.1B 15241185

[122] Stenzel-Poore MP Stevens SL Xiong Z Lessov NS Harrington CA Mori M. Effect of ischaemic preconditioning on genomic response to cerebral ischaemia: similarity to neuroprotective strategies in hibernation and hypoxia-tolerant states. Lancet. (2003) 362:1028–37. 10.1016/S0140-6736(03)14412-1 14522533

[123] Jones NM Bergeron M. Hypoxic preconditioning induces changes in HIF-1 target genes in neonatal rat brain. J Cereb Blood Flow Metab. (2001) 21:1105–14. 10.1097/00004647-200109000-00008 11524615

[124] Bernaudin M Nedelec AS Divoux D MacKenzie ET Petit E Schumann-Bard P. Normobaric hypoxia induces tolerance to focal permanent cerebral ischemia in association with an increased expression of hypoxia-inducible factor-1 and its target genes, erythropoietin and VEGF, in the adult mouse brain. J Cereb Blood Flow Metab. (2002) 22:393–403. 10.1097/00004647-200204000-00003 11919510

[125] Lee JC Tae HJ Kim IH Cho JH Lee TK Park JH. Roles of HIF-1α, VEGF, and NF-κB in ischemic preconditioning-mediated neuroprotection of hippocampal CA1 pyramidal neurons against a subsequent transient cerebral ischemia. Mol Neurobiol. (2017) 54:6984–98. 10.1007/s12035-016-0219-2 27785755

[126] Shi LB Huang JH Han BS. Hypoxia inducible factor-1alpha mediates protective effects of ischemic preconditioning on ECV-304 endothelial cells. World J Gastroenterol. (2007) 13:2369–73. 10.3748/wjg.v13.i16.2369 17511040 PMC4147150

[127] Giusti S Fiszer de Plazas S. Neuroprotection by hypoxic preconditioning involves upregulation of hypoxia-inducible factor-1 in a prenatal model of acute hypoxia. J Neurosci Res. (2012) 90:468–78. 10.1002/jnr.22766 21953610

[128] Peng Z Ren P Kang Z Du J Lian Q Liu Y. Up-regulated HIF-1alpha is involved in the hypoxic tolerance induced by hyperbaric oxygen preconditioning. Brain Res. (2008) 1212:71–8. 10.1016/j.brainres.2008.03.027 18439571

[129] Helton R Cui J Scheel JR Ellison JA Ames C Gibson C. Brain-specific knock-out of hypoxia-inducible factor-1alpha reduces rather than increases hypoxic-ischemic damage. J Neurosci. (2005) 25:4099–107. 10.1523/JNEUROSCI.4555-04.2005 15843612 PMC6724950

[130] Wang C Weihrauch D Schwabe DA Bienengraeber M Warltier DC Kersten JR. Extracellular signal-regulated kinases trigger isoflurane preconditioning concomitant with upregulation of hypoxia-inducible factor-1alpha and vascular endothelial growth factor expression in rats. Anesth Analg. (2006) 103:281–8. 10.1213/01.ane.0000226094.94877.98 16861403

[131] Agani F Jiang BH. Oxygen-independent regulation of HIF-1: novel involvement of PI3K/AKT/mTOR pathway in cancer. Curr Cancer Drug Targets. (2013) 13:245–51. 10.2174/1568009611313030003 23297826

[132] Hirayama Y Ikeda-Matsuo Y Notomi S Enaida H Kinouchi H Koizumi S. Astrocyte-mediated ischemic tolerance. J Neurosci. (2015) 35:3794–805. 10.1523/JNEUROSCI.4218-14.2015 25740510 PMC6605570

[133] Mark LP Prost RW Ulmer JL Smith MM Daniels DL Strottmann JM. Pictorial review of glutamate excitotoxicity: fundamental concepts for neuroimaging. Am J Neuroradiol. (2001) 22:1813–24. 11733308 PMC7973850

[134] Hazell AS. Excitotoxic mechanisms in stroke: an update of concepts and treatment strategies. Neurochem Int. (2007) 50:941–53. 10.1016/j.neuint.2007.04.026 17576023

[135] Paschen W. Glutamate excitotoxicity in transient global cerebral ischemia. Acta neurobiologiae experimentalis. Acta Neurobiol Exp. (1996) 56:313–22. 8787192 10.55782/ane-1996-1136

[136] Zhang J Benveniste H Klitzman B Piantadosi CA. Nitric oxide synthase inhibition and extracellular glutamate concentration after cerebral ischemia/reperfusion. Stroke. (1995) 26:298–304. 10.1161/01.STR.26.2.298 7530389

[137] Watters O O'Connor JJ. A role for tumor necrosis factor-α in ischemia and ischemic preconditioning. J Neuroinflamm. (2011) 8:87. 10.1186/1742-2094-8-87 21810263 PMC3161872

[138] Shpargel KB Jalabi W Jin Y Dadabayev A Penn MS Trapp BD. Preconditioning paradigms and pathways in the brain. Clevel Clin J Med. (2008) S77–82. 10.3949/ccjm.75.Suppl_2.S77 18540152

[139] Navon H Bromberg Y Sperling O Shani E. Neuroprotection by NMDA preconditioning against glutamate cytotoxicity is mediated through activation of ERK 1/2, inactivation of JNK, and by prevention of glutamate-induced CREB inactivation. J Mol Neurosci. (2012) 46:100–8. 10.1007/s12031-011-9532-4 21556733

[140] Soriano FX Papadia S Hofmann F Hardingham NR Bading H Hardingham GE. Preconditioning doses of NMDA promote neuroprotection by enhancing neuronal excitability. J Neurosci. (2006) 26:4509–18. 10.1523/JNEUROSCI.0455-06.2006 16641230 PMC2561857

[141] Miao B Yin XH Pei DS Zhang QG Zhang GY. Neuroprotective effects of preconditioning ischemia on ischemic brain injury through down-regulating activation of JNK1/2 via N-methyl-D-aspartate receptor-mediated Akt1 activation. J Biol Chem. (2005) 280:21693–9. 10.1074/jbc.M500003200 15797868

[142] Terasaki Y Sasaki T Yagita Y Okazaki S Sugiyama Y Oyama N. Activation of NR2A receptors induces ischemic tolerance through CREB signaling. J Cereb Blood Flow Metab. (2010) 30:1441–9. 10.1038/jcbfm.2010.18 20145658 PMC2949236

[143] Gong SJ Chen LY Zhang M Gong JX Ma YX Zhang JM. Intermittent hypobaric hypoxia preconditioning induced brain ischemic tolerance by up-regulating glial glutamate transporter-1 in rats. Neurochem Res. (2012) 37:527–37. 10.1007/s11064-011-0639-3 22076500

[144] Xu GP Dave KR Vivero R Schmidt-Kastner R Sick TJ Pérez-Pinzón MA. Improvement in neuronal survival after ischemic preconditioning in hippocampal slice cultures. Brain Res. (2002) 952:153–8. 10.1016/S0006-8993(02)02988-8 12376175

[145] Zhang M Gong JX Wang JL Jiang MY Li L Hu YY. p38 MAPK participates in the mediation of GLT-1 Up-regulation during the induction of brain ischemic tolerance by cerebral ischemic preconditioning. Mol Neurobiol. (2017) 54:58–71. 10.1007/s12035-015-9652-x 26732590

[146] Kardos J Szabó Z Héja L. Framing neuro-glia coupling in antiepileptic drug design. J Med Chem. (2016) 59:777–87. 10.1021/acs.jmedchem.5b00331 26372259

[147] Takeuchi H Jin S Suzuki H Doi Y Liang J Kawanokuchi J. Blockade of microglial glutamate release protects against ischemic brain injury. Exp Neurol. (2008) 214:144–6. 10.1016/j.expneurol.2008.08.001 18775425

[148] Ma D Feng L Cheng Y Xin M You J Yin X. Astrocytic gap junction inhibition by carbenoxolone enhances the protective effects of ischemic preconditioning following cerebral ischemia. J Neuroinflamm. (2018) 15:198. 10.1186/s12974-018-1230-5 29976213 PMC6034345

[149] Scorziello A Santillo M Adornetto A Dell'aversano C Sirabella R Damiano S. NO-induced neuroprotection in ischemic preconditioning stimulates mitochondrial Mn-SOD activity and expression via Ras/ERK1/2 pathway. J Neurochem. (2007) 103:1472–80. 10.1111/j.1471-4159.2007.04845.x 17680990

[150] Centeno JM Orti M Salom JB Sick TJ Pérez-Pinzón MA. Nitric oxide is involved in anoxic preconditioning neuroprotection in rat hippocampal slices. Brain Res. (1999) 836:62–9. 10.1016/S0006-8993(99)01610-8 10415405

[151] Atochin DN Clark J Demchenko IT Moskowitz MA Huang PL. Rapid cerebral ischemic preconditioning in mice deficient in endothelial and neuronal nitric oxide synthases. Stroke. (2003) 34:1299–303. 10.1161/01.STR.0000066870.70976.57 12677017

[152] Zhao P Zuo Z. Isoflurane preconditioning induces neuroprotection that is inducible nitric oxide synthase-dependent in neonatal rats. Anesthesiology. (2004) 101:695–703. 10.1097/00000542-200409000-00018 15329594

[153] Kapinya KJ Löwl D Fütterer C Maurer M Waschke KF Isaev NK. Tolerance against ischemic neuronal injury can be induced by volatile anesthetics and is inducible NO synthase dependent. Stroke. (2002) 33:1889–98. 10.1161/01.STR.0000020092.41820.58 12105371

[154] Vellimana AK Milner E Azad TD Harries MD Zhou ML Gidday JM. Endothelial nitric oxide synthase mediates endogenous protection against subarachnoid hemorrhage-induced cerebral vasospasm. Stroke. (2011) 42:776–82. 10.1161/STROKEAHA.110.607200 21317271 PMC3042520

[155] Wang Q Tang XN Yenari MA. The inflammatory response in stroke. J Neuroimmunol. (2007) 184:53–68. 10.1016/j.jneuroim.2006.11.014 17188755 PMC1868538

[156] Gesuete R Stevens SL Stenzel-Poore MP. Role of circulating immune cells in stroke and preconditioning-induced protection. Acta Neurochirurgica Suppl. (2016) 121:39–44. 10.1007/978-3-319-18497-5_7 26463920 PMC4664184

[157] Terao S Yilmaz G Stokes KY Ishikawa M Kawase T Granger DN. Inflammatory and injury responses to ischemic stroke in obese mice. Stroke. (2008) 39:943–50. 10.1161/STROKEAHA.107.494542 18239178

[158] Jin R Yang G Li G. Inflammatory mechanisms in ischemic stroke: role of inflammatory cells. J Leukocyte Biol. (2010) 87:779–89. 10.1189/jlb.1109766 20130219 PMC2858674

[159] Wang YC Lin S Yang QW Toll-like receptors in cerebral ischemic inflammatory injury. J Neuroinflamm. (2011) 8:134 10.1186/1742-2094-8-134 PMC3198933 21982558

[160] Pan LN Zhu W Li Y Xu XL Guo LJ Lu Q. Astrocytic Toll-like receptor 3 is associated with ischemic preconditioning-induced protection against brain ischemia in rodents. PLoS ONE. (2014) 9:e99526. 10.1371/journal.pone.0099526 24914679 PMC4051824

[161] Stevens SL Ciesielski TM Marsh BJ Yang T Homen DS Boule JL. Toll-like receptor 9: a new target of ischemic preconditioning in the brain. J Cereb Blood Flow Metab. (2008) 28:1040–7. 10.1038/sj.jcbfm.9600606 18183029 PMC3037270

[162] Pradillo JM Fernández-López D García-Yébenes I Sobrado M Hurtado O Moro MA. Toll-like receptor 4 is involved in neuroprotection afforded by ischemic preconditioning. J Neurochem. (2009) 109:287–94. 10.1111/j.1471-4159.2009.05972.x 19200341

[163] Wang PF Xiong XY Chen J Wang YC Duan W Yang QW. Function and mechanism of toll-like receptors in cerebral ischemic tolerance: from preconditioning to treatment. J Neuroinflamm. (2015) 12:80. 10.1186/s12974-015-0301-0 25928750 PMC4422156

[164] Stevens SL Leung PY Vartanian KB Gopalan B Yang T Simon RP. Multiple preconditioning paradigms converge on interferon regulatory factor-dependent signaling to promote tolerance to ischemic brain injury. J Neurosci. (2011) 31:8456–63. 10.1523/JNEUROSCI.0821-11.2011 21653850 PMC3130521

[165] Pérez-Pinzón MA Vitro TM Dietrich WD Sick TJ. The effect of rapid preconditioning on the microglial, astrocytic and neuronal consequences of global cerebral ischemia. Acta Neuropathol. (1999) 97:495–501. 10.1007/s004010051019 10334487

[166] Pérez-Pinzón MA Xu GP Mumford PL Dietrich WD Rosenthal M Sick TJ. Rapid ischemic preconditioning protects rats from cerebral anoxia/ischemia. Adv Exp Med Biol. (1997) 428:155–61. 10.1007/978-1-4615-5399-1_22 9500042

[167] Nakamura M Nakakimura K Matsumoto M Sakabe T. Rapid tolerance to focal cerebral ischemia in rats is attenuated by adenosine A1 receptor antagonist. J Cereb Blood Flow Metab. (2002) 22:161–70. 10.1097/00004647-200202000-00004 11823714

[168] Caparrelli DJ Cattaneo SM Bethea BT Shake JG Eberhart C Blue ME. Pharmacological preconditioning ameliorates neurological injury in a model of spinal cord ischemia. Annals Thoracic Surg. (2002) 74:838–44; discussion 844–5. 10.1016/S0003-4975(02)03716-5 12238848

[169] Rehni AK Singh TG. Involvement of CCR-2 chemokine receptor activation in ischemic preconditioning and postconditioning of brain in mice. Cytokine. (2012) 60:83–9. 10.1016/j.cyto.2012.05.009 22704692

[170] Dong H Xiong L Zhu Z Chen S Hou L Sakabe T. Preconditioning with hyperbaric oxygen and hyperoxia induces tolerance against spinal cord ischemia in rabbits. Anesthesiology. (2002) 96:907–12. 10.1097/00000542-200204000-00018 11964598

[171] Nogawa S Zhang F Ross ME Iadecola C. Cyclo-oxygenase-2 gene expression in neurons contributes to ischemic brain damage. J Neurosci. (1997) 17:2746–55. 10.1523/JNEUROSCI.17-08-02746.1997 9092596 PMC6573095

[172] Colangelo V Gordon WC Mukherjee PK Trivedi P Ottino P. Downregulation of COX-2 and JNK expression after induction of ischemic tolerance in the gerbil brain. Brain Res. (2004) 1016:195–200. 10.1016/j.brainres.2004.05.017 15246855

[173] Lee JC Tae HJ Cho GS Kim IH Ahn JH Park JH. Ischemic preconditioning protects neurons from damage and maintains the immunoreactivity of kynurenic acid in the gerbil hippocampal CA1 region following transient cerebral ischemia. Int J Mol Med. (2015) 35:1537–44. 10.3892/ijmm.2015.2171 25872573 PMC4432926

[174] Shen HY Lusardi TA Williams-Karnesky RL Lan JQ Poulsen DJ Boison D. Adenosine kinase determines the degree of brain injury after ischemic stroke in mice. J Cereb Blood Flow Metab. (2011) 31:1648–59. 10.1038/jcbfm.2011.30 21427729 PMC3137468

[175] Hong S Ahn JY Cho GS Kim IH Cho JH Ahn JH. Monocarboxylate transporter 4 plays a significant role in the neuroprotective mechanism of ischemic preconditioning in transient cerebral ischemia. Neural Regenerat Res. (2015) 10:1604–11. 10.4103/1673-5374.167757 26692857 PMC4660753

[176] Tajiri S Oyadomari S Yano S Morioka M Gotoh T Hamada JI. Ischemia-induced neuronal cell death is mediated by the endoplasmic reticulum stress pathway involving CHOP. Cell Death Different. (2004) 11:403–15. 10.1038/sj.cdd.4401365 14752508

[177] Hayashi T Saito A Okuno S Ferrand-Drake M Dodd RL Chan PH. Damage to the endoplasmic reticulum and activation of apoptotic machinery by oxidative stress in ischemic neurons. J Cereb Blood Flow Metab. (2005) 25:41–53. 10.1038/sj.jcbfm.9600005 15678111

[178] Hu YQ Chen W Yan MH Lai JJ Tang N Wu L. Ischemic preconditioning protects brain from ischemia/reperfusion injury by attenuating endoplasmic reticulum stress-induced apoptosis through PERK pathway. Eur Rev Med Pharmacol Sci. (2017) 21:5736–44. 10.26355/eurrev_201712_14020 29272010

[179] Venna VR Li J Benashski SE Tarabishy S McCullough LD. Preconditioning induces sustained neuroprotection by downregulation of adenosine 5'-monophosphate-activated protein kinase. Neuroscience. (2012) 201:280–7. 10.1016/j.neuroscience.2011.11.014 22120436 PMC3258333

[180] Pignataro G Boscia F Esposito E Sirabella R Cuomo O Vinciguerra A. NCX1 and NCX3: two new effectors of delayed preconditioning in brain ischemia. Neurobiol Dis. (2012) 45:616–23. 10.1016/j.nbd.2011.10.007 22036625

[181] Yan W Fang Z Yang Q Dong H Lu Y Lei C. SirT1 mediates hyperbaric oxygen preconditioning-induced ischemic tolerance in rat brain. J Cereb Blood Flow Metab. (2013) 33:396–406. 10.1038/jcbfm.2012.179 23299244 PMC3587810

[182] Mathew R Khor S Hackett SR Rabinowitz JD Perlman DH White E. Functional role of autophagy-mediated proteome remodeling in cell survival signaling and innate immunity. Mol Cell. (2014) 55:916–30. 10.1016/j.molcel.2014.07.019 25175026 PMC4169768

[183] Codogno P Meijer AJ Autophagy and signaling: their role in cell survival and cell death. Cell Death Different. (2005) 1509–18. 10.1038/sj.cdd.4401751 16247498

[184] Carloni S Buonocore G Balduini W. Protective role of autophagy in neonatal hypoxia-ischemia induced brain injury. Neurobiol Dis. (2008) 32:329–39. 10.1016/j.nbd.2008.07.022 18760364

[185] Sheng R Zhang LS Han R Liu XQ Gao B Qin ZH Autophagy activation is associated with neuroprotection in a rat model of focal cerebral ischemic preconditioning. Autophagy. (2010) 6:482–94. 10.4161/auto.6.4.11737 20400854

[186] Cho BB Toledo-Pereyra LH. Caspase-independent programmed cell death following ischemic stroke. J Investigat Surg. (2008) 21:141–7. 10.1080/08941930802029945 18569435

[187] Xia DY Li W Qian HR Yao S Liu JG Qi XK. Ischemia preconditioning is neuroprotective in a rat cerebral ischemic injury model through autophagy activation and apoptosis inhibition. Brazil J Med Biol Res. (2013) 46:580–8. 10.1590/1414-431X20133161 23903681 PMC3859329

[188] Jordan J Böttner M Schluesener HJ Unsicker K Krieglstein K. Bone morphogenetic proteins: neurotrophic roles for midbrain dopaminergic neurons and implications of astroglial cells. Eur J Neurosci. (1997) 9:1699–709. 10.1111/j.1460-9568.1997.tb01527.x 9283824

[189] Guan J Li H Lv T Chen D Yuan Y Qu S. Bone morphogenetic protein-7 (BMP-7) mediates ischemic preconditioning-induced ischemic tolerance via attenuating apoptosis in rat brain. Biochem Biophys Res Commun. (2013) 441:560–6. 10.1016/j.bbrc.2013.10.121 24184479

[190] Guan J Li H Lv T Chen D Yuan Y Qu S. Bone morphogenic protein-7 contributes to cerebral ischemic preconditioning induced-ischemic tolerance by activating p38 mitogen-activated protein kinase signaling pathway. Inflammation. (2014) 37:1289–96. 10.1007/s10753-014-9856-7 24682853

[191] Suh SW Gum ET Hamby AM Chan PH Swanson RA. Hypoglycemic neuronal death is triggered by glucose reperfusion and activation of neuronal NADPH oxidase. J Clin Investig. (2007) 117:910–8. 10.1172/JCI30077 17404617 PMC1838937

[192] Tohyama Y Sako K Yonemasu Y. Hypothermia attenuates hyperglycolysis in the periphery of ischemic core in rat brain. Exp Brain Res. (1998) 122:333–8. 10.1007/s002210050521 9808306

[193] Parsons MW Barber PA Desmond PM Baird TA Darby DG Byrnes G. Acute hyperglycemia adversely affects stroke outcome: a magnetic resonance imaging and spectroscopy study. Annals Neurol. (2002) 52:20–8. 10.1002/ana.10241 12112043

[194] Geng J Zhang Y Li S Li S Wang J Wang H. Metabolomic profiling reveals that reprogramming of cerebral glucose metabolism is involved in ischemic preconditioning-induced neuroprotection in a rodent model of ischemic stroke. J Proteome Res. (2019) 18:57–68. 10.1021/acs.jproteome.8b00339 30362349

[195] Wang B Cao W Biswal S Doré S. Carbon monoxide-activated Nrf2 pathway leads to protection against permanent focal cerebral ischemia. Stroke. (2011) 42:2605–10. 10.1161/STROKEAHA.110.607101 21852618 PMC3278075

[196] Bell KF Fowler JH Al-Mubarak B Horsburgh K Hardingham GE. Activation of Nrf2-regulated glutathione pathway genes by ischemic preconditioning. Oxidative Med Cell Longevity. (2011) 2011:689524. 10.1155/2011/689524 21904646 PMC3166574

[197] Narayanan SV Dave KR Perez-Pinzon MA. Ischemic preconditioning protects astrocytes against oxygen glucose deprivation via the nuclear erythroid 2-related factor 2 pathway. Transl Stroke Res. (2018) 9:99–109. 10.1007/s12975-017-0574-y 29103101 PMC6771255

[198] Park JH Lee CH Kim IH Ahn JH Cho JH Yan BC. Time-course changes in immunoreactivities of glucokinase and glucokinase regulatory protein in the gerbil hippocampus following transient cerebral ischemia. Neurochem Res. (2013) 38:2640–9. 10.1007/s11064-013-1182-1 24146201

[199] Cho YS Cho JH Shin BN Cho GS Kim IH Park JH. Ischemic preconditioning maintains the immunoreactivities of glucokinase and glucokinase regulatory protein in neurons of the gerbil hippocampal CA1 region following transient cerebral ischemia. Mol Med Rep. (2015) 12:4939–46. 10.3892/mmr.2015.4021 26134272 PMC4581829

[200] Andjelkovic AV Stamatovic SM Keep RF. The protective effects of preconditioning on cerebral endothelial cells *in vitro*. J Cereb Blood Flow Metab. (2003) 23:1348–55. 10.1097/01.WCB.0000091762.61714.FE 14600442

[201] Shi Y Zhang L Pu H Mao L Hu X Jiang X. Rapid endothelial cytoskeletal reorganization enables early blood-brain barrier disruption and long-term ischaemic reperfusion brain injury. Nat Commun. (2016) 7:10523. 10.1038/ncomms10523 26813496 PMC4737895

[202] Wacker BK Freie AB Perfater JL Gidday JM. Junctional protein regulation by sphingosine kinase 2 contributes to blood-brain barrier protection in hypoxic preconditioning-induced cerebral ischemic tolerance. J Cereb Blood Flow Metab. (2012) 32:1014–23. 10.1038/jcbfm.2012.3 22314269 PMC3367228

[203] Zhang FY Chen XC Ren HM Bao WM. Effects of ischemic preconditioning on blood-brain barrier permeability and MMP-9 expression of ischemic brain. Neurol Res. (2006) 28:21–4. 10.1179/016164106X91825 16464358

[204] Nazari M Keshavarz S Rafati A Namavar MR Haghani M. Fingolimod (FTY720) improves hippocampal synaptic plasticity and memory deficit in rats following focal cerebral ischemia. Brain Res Bull. (2016) 124:95–102. 10.1016/j.brainresbull.2016.04.004 27066884

[205] Haghani M Keshavarz S Nazari M Rafati A. Electrophysiology of cerebral ischemia and reperfusion: first evidence for the role of synapse in ischemic tolerance. Synapse. (2016) 70:351–60. 10.1002/syn.21910 27124112

[206] Bigdeli MR Khoshbaten A. *In vivo* preconditioning with normobaric hyperoxia induces ischemic tolerance partly by triggering tumor necrosis factor-alpha converting enzyme/tumor necrosis factor-alpha/nuclear factor-kappaB. Neuroscience. (2008) 153:671–8. 10.1016/j.neuroscience.2008.02.064 18423996

[207] Chavez JC Baranova O Lin J Pichiule P. The transcriptional activator hypoxia inducible factor 2 (HIF-2/EPAS-1) regulates the oxygen-dependent expression of erythropoietin in cortical astrocytes. J Neurosci. (2006) 26:9471–81. 10.1523/JNEUROSCI.2838-06.2006 16971531 PMC6674587

[208] Vangeison G Rempe DA. The Janus-faced effects of hypoxia on astrocyte function. Neuroscientist. (2009) 15:579–88. 10.1177/1073858409332405 19359669 PMC2801159

[209] Huang CY Fujimura M Chang YY Chan PH. Overexpression of copper-zinc superoxide dismutase attenuates acute activation of activator protein-1 after transient focal cerebral ischemia in mice. Stroke. (2001) 32:741–7. 10.1161/01.STR.32.3.741 11239196

[210] Kapinya K Penzel R Sommer C Kiessling M. Temporary changes of the AP-1 transcription factor binding activity in the gerbil hippocampus after transient global ischemia, and ischemic tolerance induction. Brain Res. (2000) 872:282–93. 10.1016/S0006-8993(00)02503-8 10924710

[211] Wang LW Tu YF Huang CC Ho CJ. JNK signaling is the shared pathway linking neuroinflammation, blood-brain barrier disruption, and oligodendroglial apoptosis in the white matter injury of the immature brain. J Neuroinflamm. (2012) 9:175. 10.1186/1742-2094-9-175 22805152 PMC3414763

[212] Zhan L Wang T Li W Xu ZC Sun W Xu E. Activation of Akt/FoxO signaling pathway contributes to induction of neuroprotection against transient global cerebral ischemia by hypoxic pre-conditioning in adult rats. J Neurochem. (2010) 114:897–908. 10.1111/j.1471-4159.2010.06816.x 20492357

[213] Dhodda VK Sailor KA Bowen KK Vemuganti R. Putative endogenous mediators of preconditioning-induced ischemic tolerance in rat brain identified by genomic and proteomic analysis. J Neurochem. (2004) 89:73–89. 10.1111/j.1471-4159.2004.02316.x 15030391

[214] Lusardi TA Farr CD Faulkner CL Pignataro G Yang T Lan J. Ischemic preconditioning regulates expression of microRNAs and a predicted target, MeCP2, in mouse cortex. J Cereb Blood Flow Metab. (2010) 30:744–56. 10.1038/jcbfm.2009.253 20010955 PMC2935903

[215] Xu H Lu A Sharp FR. Regional genome transcriptional response of adult mouse brain to hypoxia. BMC Genomics. (2011) 12:499. 10.1186/1471-2164-12-499 21988864 PMC3218040

[216] Thompson JW Dave KR Young JI Perez-Pinzon MA. Ischemic preconditioning alters the epigenetic profile of the brain from ischemic intolerance to ischemic tolerance. Neurotherapeutics. (2013) 10:789–97. 10.1007/s13311-013-0202-9 23868468 PMC3805868

[217] Veighey K Macallister RJ. Clinical applications of remote ischemic preconditioning. Cardiol Res Pract. (2012) 2012:620681. 10.1155/2012/620681 22400123 PMC3286899

[218] Meng R Asmaro K Meng L Liu Y Ma C Xi C. Upper limb ischemic preconditioning prevents recurrent stroke in intracranial arterial stenosis. Neurology. (2012) 79:1853–61. 10.1212/WNL.0b013e318271f76a 23035060

[219] Walsh SR Nouraei SA Tang TY Sadat U Carpenter RH Gaunt ME. Remote ischemic preconditioning for cerebral and cardiac protection during carotid endarterectomy: results from a pilot randomized clinical trial. Vascul Endovasc Surg. (2010) 44:434–9. 10.1177/1538574410369709 20484064

[220] Røpcke DM Hjortdal VE Toft GE Jensen MO Kristensen SD. Remote ischemic preconditioning reduces thrombus formation in the rat. J Thromb Haemostasis. (2012) 10:2405–6. 10.1111/j.1538-7836.2012.04914.x 22947090

[221] Liem DA Verdouw PD Duncker DJ. Transient limb ischemia induces remote ischemic preconditioning *in vivo*. Circulation. (2003) 107:e218–9; author reply: e218–9. 10.1161/01.CIR.0000077520.36997.F9 12821595

[222] Davies WR Brown AJ Watson W McCormick LM West NE Dutka DP. Remote ischemic preconditioning improves outcome at 6 years after elective percutaneous coronary intervention: the CRISP stent trial long-term follow-up. Circ Cardiovasc Int. (2013) 6:246–51. 10.1161/CIRCINTERVENTIONS.112.000184 23696599

[223] Hu S Dong H Zhang H Wang S Hou L Chen S. Noninvasive limb remote ischemic preconditioning contributes neuroprotective effects via activation of adenosine A1 receptor and redox status after transient focal cerebral ischemia in rats. Brain Res. (2012) 1459:81–90. 10.1016/j.brainres.2012.04.017 22560096

[224] Hoda MN Bhatia K Hafez SS Johnson MH Siddiqui S Ergul A. Remote ischemic perconditioning is effective after embolic stroke in ovariectomized female mice. Transl Stroke Res. (2014) 5:484–90. 10.1007/s12975-013-0318-6 24385308 PMC4092232

[225] Ren C Gao X Steinberg GK Zhao H. Limb remote-preconditioning protects against focal ischemia in rats and contradicts the dogma of therapeutic time windows for preconditioning. Neuroscience. (2008) 151:1099–103. 10.1016/j.neuroscience.2007.11.056 18201834 PMC2696348

[226] Carter HH Maxwell JD Hellsten Y Thompson A Thijssen DHJ Jones H. The impact of acute remote ischaemic preconditioning on cerebrovascular function. Eur J Appl Physiol. (2020) 120:603–12. 10.1007/s00421-019-04297-1 31932877 PMC7042189

[227] Connolly M Bilgin-Freiert A Ellingson B Dusick JR Liebeskind D Saver J. Peripheral vascular disease as remote ischemic preconditioning, for acute stroke. Clin Neurol Neurosurg. (2013) 115:2124–9. 10.1016/j.clineuro.2013.07.038 23958050 PMC3798063

[228] Loukogeorgakis SP Panagiotidou AT Broadhead MW Donald A Deanfield JE MacAllister RJ. Remote ischemic preconditioning provides early and late protection against endothelial ischemia-reperfusion injury in humans: role of the autonomic nervous system. J Am Coll Cardiol. (2005) 46:450–6. 10.1016/j.jacc.2005.04.044 16053957

[229] Wei D Ren C Chen X Zhao H. The chronic protective effects of limb remote preconditioning and the underlying mechanisms involved in inflammatory factors in rat stroke. PLoS ONE. (2012) 7:e30892. 10.1371/journal.pone.0030892 22347410 PMC3275571

[230] Malhotra S Naggar I Stewart M Rosenbaum DM. Neurogenic pathway mediated remote preconditioning protects the brain from transient focal ischemic injury. Brain Res. (2011) 1386:184–90. 10.1016/j.brainres.2011.02.032 21338588

[231] Hess DC Hoda MN Bhatia K. Remote limb perconditioning [corrected] and postconditioning: will it translate into a promising treatment for acute stroke? Stroke. (2013) 44:1191–7. 10.1161/STROKEAHA.112.678482 23339961

[232] Shimizu M Tropak M Diaz RJ Suto F Surendra H Kuzmin E. Transient limb ischaemia remotely preconditions through a humoral mechanism acting directly on the myocardium: evidence suggesting cross-species protection. Clini Sci. (2009) 117:191–200. 10.1042/CS20080523 19175358

[233] Bonova P Gottlieb M. Blood as the carrier of ischemic tolerance in rat brain. J Neurosci Res. (2015) 93:1250–7. 10.1002/jnr.23580 25787695

[234] Kanoria S Jalan R Seifalian AM Williams R Davidson BR. Protocols and mechanisms for remote ischemic preconditioning: a novel method for reducing ischemia reperfusion injury. Transplantation. (2007) 84:445–58. 10.1097/01.tp.0000228235.55419.e8 17713425

[235] Ng MW Angerosa J Konstantinov IE Cheung MM Pepe S. Remote ischaemic preconditioning modifies serum apolipoprotein D, met-enkephalin, adenosine, and nitric oxide in healthy young adults. Clin Exp Pharmacol Physiol. (2019) 46:995–1000. 10.1111/1440-1681.13150 31361911

[236] Heusch G Musiolik J Kottenberg E Peters J Jakob H Thielmann M. STAT5 activation and cardioprotection by remote ischemic preconditioning in humans: short communication. Circ Res. (2012) 110:111–5. 10.1161/CIRCRESAHA.111.259556 22116817

[237] Mastitskaya S Marina N Gourine A Gilbey MP Spyer KM Teschemacher AG. Cardioprotection evoked by remote ischaemic preconditioning is critically dependent on the activity of vagal pre-ganglionic neurones. Cardiovasc Res. (2012) 95:487–94. 10.1093/cvr/cvs212 22739118 PMC3422080

[238] Sun Z Baker W Hiraki T Greenberg JH. The effect of right vagus nerve stimulation on focal cerebral ischemia: an experimental study in the rat. Brain Stimul. (2012) 5:1–10. 10.1016/j.brs.2011.01.009 22037134 PMC3264742

[239] Donato M Buchholz B Rodríguez M Pérez V Inserte J García-Dorado D. Role of the parasympathetic nervous system in cardioprotection by remote hindlimb ischaemic preconditioning. Exp Physiol. (2013) 98:425–34. 10.1113/expphysiol.2012.066217 22872660

[240] Steensrud T Li J Dai X Manlhiot C Kharbanda RK Tropak M Pretreatment with the nitric oxide donor SNAP or nerve transection blocks humoral preconditioning by remote limb ischemia or intra-arterial adenosine. Am J Physiol Heart Circ Physiol. (2010) 299:H1598–603. 10.1152/ajpheart.00396.2010 20802131

[241] Rassaf T Totzeck M Hendgen-Cotta UB Shiva S Heusch G Kelm M. Circulating nitrite contributes to cardioprotection by remote ischemic preconditioning. Circ Res. (2014) 114:1601–10. 10.1161/CIRCRESAHA.114.303822 24643960

[242] Lim SY Yellon DM Hausenloy DJ. The neural and humoral pathways in remote limb ischemic preconditioning. Basic Res Cardiol. (2010) 105:651–5. 10.1007/s00395-010-0099-y 20449597

[243] Redington KL Disenhouse T Strantzas SC Gladstone R Wei C Tropak MB. Remote cardioprotection by direct peripheral nerve stimulation and topical capsaicin is mediated by circulating humoral factors. Basic Res Cardiol. (2012) 107:241. 10.1007/s00395-011-0241-5 22231674

[244] Konstantinov IE Arab S Kharbanda RK Li J Cheung MM Cherepanov V. The remote ischemic preconditioning stimulus modifies inflammatory gene expression in humans. Physiol Genomics. (2004) 19:143–50. 10.1152/physiolgenomics.00046.2004 15304621

[245] Shimizu M Saxena P Konstantinov IE Cherepanov V Cheung MM Wearden P. Remote ischemic preconditioning decreases adhesion and selectively modifies functional responses of human neutrophils. J Surg Res. (2010) 158:155–61. 10.1016/j.jss.2008.08.010 19540519

[246] Liu ZJ Chen C Li XR Ran YY Xu T Zhang Y. Remote ischemic preconditioning-mediated neuroprotection against stroke is associated with significant alterations in peripheral immune responses. CNS Neurosci Therapeut. (2016) 22:43–52. 10.1111/cns.12448 26384716 PMC6492849

[247] Weber C. Far from the heart: receptor cross-talk in remote conditioning. Nat Med. (2010) 16:760–2. 10.1038/nm0710-760 20613755

[248] Fan J Zhang Z Chao X Gu J Cai W Zhou W. Ischemic preconditioning enhances autophagy but suppresses autophagic cell death in rat spinal neurons following ischemia-reperfusion. Brain Res. (2014) 1562:76–86. 10.1016/j.brainres.2014.03.019 24675029

[249] Park HK Chu K Jung KH Lee ST Bahn JJ Kim M. Autophagy is involved in the ischemic preconditioning. Neurosci Lett. (2009) 451:16–9. 10.1016/j.neulet.2008.12.019 19103253

[250] Lv J Guan W You Q Deng L Zhu Y Guo K. RIPC provides neuroprotection against ischemic stroke by suppressing apoptosis via the mitochondrial pathway. Sci Rep. (2020) 10:5361. 10.1038/s41598-020-62336-w 32210331 PMC7093414

[251] Raval AP Bramlett H Perez-Pinzon MA. Estrogen preconditioning protects the hippocampal CA1 against ischemia. Neuroscience. (2006) 141:1721–30. 10.1016/j.neuroscience.2006.05.016 16777351

[252] Kim JH Choi KH Jang YJ Kim HN Bae SS Choi BT. Electroacupuncture preconditioning reduces cerebral ischemic injury via BDNF and SDF-1α in mice. BMC Compl Alternat Med. (2013) 13:22. 10.1186/1472-6882-13-22 23356671 PMC3562247

[253] Yang Q Guo M Wang X Zhao Y Zhao Q Ding H. Ischemic preconditioning with a ketogenic diet improves brain ischemic tolerance through increased extracellular adenosine levels and hypoxia-inducible factors. Brain Res. (2017) 1667:11–18. 10.1016/j.brainres.2017.04.010 28427869

[254] Liebelt B Papapetrou P Ali A Guo M Ji X Peng C. Exercise preconditioning reduces neuronal apoptosis in stroke by up-regulating heat shock protein-70 (heat shock protein-72) and extracellular-signal-regulated-kinase 1/2. Neuroscience. (2010) 166:1091–100. 10.1016/j.neuroscience.2009.12.067 20083167

[255] Lee HI Park JH Park MY Kim NG Park KJ Choi BT. Pre-conditioning with transcranial low-level light therapy reduces neuroinflammation and protects blood-brain barrier after focal cerebral ischemia in mice. Restorat Neurol Neurosci. (2016) 34:201–14. 10.3233/RNN-150559 26889965

[256] Kepler T Kuusik K Lepner U Starkopf J Zilmer M Eha J. Remote ischaemic preconditioning attenuates cardiac biomarkers during vascular surgery: a randomised clinical trial. Eur J Vascul Endovascul Surg. (2020) 59:301–8. 10.1016/j.ejvs.2019.09.502 31870692

[257] Oh CS Sa M Park HJ Piao L Oh KS Kim SH. Effects of remote ischemic preconditioning on regional cerebral oxygen saturation in patients in the beach chair position during shoulder surgery: a double-blind randomized controlled trial. J Clin Anesthesia. (2020) 61:109661. 10.1016/j.jclinane.2019.109661 31818636

[258] Moskowitz MA Waeber C. Remote ischemic preconditioning: making the brain more tolerant, safely and inexpensively. Circulation. (2011) 123:709–11. 10.1161/CIRCULATIONAHA.110.009688 21300956

[259] Vasdekis SN Athanasiadis D Lazaris A Martikos G Katsanos AH Tsivgoulis G. The role of remote ischemic preconditioning in the treatment of atherosclerotic diseases. Brain Behav. (2013) 3:606–16. 10.1002/brb3.161 24363964 PMC3868166

[260] Koch S Gonzalez N. Preconditioning the human brain: proving the principle in subarachnoid hemorrhage. Stroke. (2013) 44:1748–53. 10.1161/STROKEAHA.111.000773 23598525

[261] Gonzalez NR Hamilton R Bilgin-Freiert A Dusick J Vespa P Hu X. Cerebral hemodynamic and metabolic effects of remote ischemic preconditioning in patients with subarachnoid hemorrhage. Acta Neurochirurg Suppl. (2013) 115:193–8. 10.1007/978-3-7091-1192-5_36 22890668

[262] Zuo Z Are volatile anesthetics neuroprotective or neurotoxic? Med Gas Res. (2012) 2:10 10.1186/2045-9912-2-10 22510328 PMC3353836

